# Respiratory viral co‐infections in patients with COVID‐19 and associated outcomes: A systematic review and meta‐analysis

**DOI:** 10.1002/rmv.2365

**Published:** 2022-06-10

**Authors:** Hanna Krumbein, Lara S. Kümmel, Paraskevi C. Fragkou, Clemens Thölken, Ben L. Hünerbein, Rieke Reiter, Konstantinos A. Papathanasiou, Harald Renz, Chrysanthi Skevaki

**Affiliations:** ^1^ Institute of Laboratory Medicine Universities of Giessen and Marburg Lung Center (UGMLC) Philipps Universität Marburg German Center for Lung Research (DZL) Marburg Marburg Germany; ^2^ Fourth Department of Internal Medicine Medical School of Athens National and Kapodistrian University of Athens Attikon University Hospital Athens Greece; ^3^ Institute of Medical Bioinformatics and Biostatistics Medical Faculty Philipps University of Marburg Marburg Germany; ^4^ Medical School National and Kapodistrian University of Athens Athens Greece

**Keywords:** co‐infection, COVID‐19, meta‐analysis, respiratory viruses, SARS‐CoV‐2

## Abstract

The aim of this systematic review and meta‐analysis was to critically assess the published literature related to community‐acquired viral co‐infections and COVID‐19 and to evaluate the prevalence, most identified co‐pathogens, and relevant risk factors. Furthermore, we aimed to examine the clinical features and outcomes of co‐infected compared to mono‐infected COVID‐19 patients. We systematically searched PubMed, Web of Science, Embase, Scopus, and The Cochrane Library for studies published from 1 November 2019 to 13 August 2021. We included patients of all ages and any COVID‐19 severity who were screened for respiratory viral co‐infection within 48 h of COVID‐19 diagnosis. The main outcome was the proportion of patients with a respiratory viral co‐infection. The systematic review was registered to PROSPERO (CRD42021272235). Out of 6053 initially retrieved studies, 59 studies with a total of 16,643 SARS‐CoV‐2 positive patients were included. The global pooled prevalence was 5.01% (95% CI 3.34%–7.27%; *I* ^*2*^ = 95%) based on a random‐effects model, with Influenza Viruses (1.54%) and Enteroviruses (1.32%) being the most prevalent pathogens. Subgroup analyses showed that co‐infection was significantly higher in paediatric (9.39%) than adult (3.51%) patients (*p*‐value = 0.02). Furthermore, co‐infected patients were more likely to be dyspnoeic and the odds of fatality (OR = 1.66) were increased. Although a relatively low proportion of COVID‐19 patients have a respiratory viral co‐infection, our findings show that multiplex viral panel testing may be advisable in patients with compatible symptoms. Indeed, respiratory virus co‐infections may be associated with adverse clinical outcomes and therefore have therapeutic and prognostic implications.

**Abbreviations**
FLU: Influenza Virus
HAdV: human adenovirus
HBoV: human bocavirus
HCoV: common human coronavirus
HPIV: human parainfluenza virus
HRSV: human respiratory syncytial virus
MERS‐CoV: Middle East respiratory syndrome‐related coronavirus
NOS: Newcastle‐Ottawa Scale
OR: Odds ratios
PRISMA: Preferred Reporting Items for Systematic Reviews and Meta‐Analyses
QoE: quality of evidence
RoB: Risk of publication bias
RT‐PCR: reverse‐transcription polymerase chain reaction
RV: human rhinoviruses
RVCI: respiratory viral co‐infection
SARS‐CoV: severe acute respiratory coronavirus
WHO: World Health Organisation

## 1 INTRODUCTION

In December 2019 a novel virus, severe acute respiratory coronavirus 2 (SARS‐CoV‐2) was first described in Wuhan, China. Since then, the enveloped RNA betacoronavirus has spread across the world and is currently associated a global pandemic as declared by the World Health Organization (WHO) in March 2020.^1^ Since the onset of the pandemic, an immense number of studies has been performed to comprehend the disease and its pathophysiology as well as to generate new therapeutic approaches.^2^ Co‐occurrence of respiratory infections may be one of the factors that leads to an increased disease severity.^3^

Due to their mode of transmission, which is mainly by droplets, respiratory virus circulation within the community is high and dual infections within their realm are widespread. In the pre‐COVID‐19 pandemic era a 10th of respiratory virus infections was found to be co‐infected with another respiratory virus.^4^ Various studies observed an increased severity of disease in co‐infected patients, especially elderly and high‐risk patients.^5^

While several studies have reported the co‐detection of SARS‐CoV‐2 with additional respiratory viruses, questions remain regarding the clinical relevance. Virus‐virus interaction (either direct or immune‐mediated) can have effects on disease severity, transmissibility, immunopathology, and vaccine effectiveness. With SARS‐CoV‐2, including its variants, becoming firmly established in the human population, it is important to investigate the possible consequences of respiratory viral co‐infections (RVCI).^6^, ^7^

Data on the prevalence and the most common co‐infecting viruses will help clinicians to implement appropriate infection control measures and treat patients adequately, including administering an adequate antiviral therapy whenever available and appropriate. Knowledge on risk factors for co‐infection and on the possible changes in terms of clinical progression of the disease is important to assess the patients' prognosis.

The aim of the present systematic review was to critically assess the published literature related to community‐acquired viral co‐infections and COVID‐19 and to evaluate the prevalence, most identified co‐pathogens, and relevant risk factors. Furthermore, we aimed to examine the clinical features and outcomes of RVCI compared to mono‐infected COVID‐19 patients.

## 2 METHODS

This systematic review followed the Preferred Reporting Items for Systematic Reviews and Meta‐Analyses (PRISMA) guidelines^8^ (Table S6) and was registered to PROSPERO (registration number: CRD42021272235).

### 2.1 Search strategy and selection criteria

PubMed, Web of Science, Embase, Scopus, and The Cochrane Library were systematically searched for studies published between 1 November 2019 and 13 August 2021 in the English or German language. Search terms were combinations of three concepts comprising of COVID‐19 related words (e.g., ‘SARS‐CoV‐2, ‘2019 nCoV’), co‐infection related terms (e.g., ‘concurrent infection’, ‘dual infection’), and the names and its variants of each of the 18 included respiratory viral co‐pathogens. A complete description of our search strings is available in Table S1. The inclusion criteria for the studies included (i) COVID‐19 diagnosis according to the WHO COVID‐19 case definition^9^; (ii) patients of any age, setting and severity of illness; (iii) a test for co‐infection with any of 18 predefined respiratory viruses within 48 h of COVID‐19 diagnosis. Publications that were excluded included: (i) case reports and case series with less than 10 participants, (ii) reviews, (iii) conference abstracts and (iv) studies thematically unrelated to the study objective (Table S2).

### 2.2 Data extraction

Four independent reviewers (Hanna Krumbein, Lara S. Kümmel, Ben L. Hünerbein, Rieke Reiter) screened the titles and abstracts to identify potentially eligible studies. The same reviewers screened the full texts of the possibly relevant studies. Disagreements among reviewers were resolved by a fifth independent reviewer (Konstantinos A. Papathanasiou). Two reviewers (Hanna Krumbein, Lara S. Kümmel) independently extracted data from the individual studies using a predefined template. Discrepancies were resolved by discussion between the two reviewers. The extracted data included information regarding the study itself (authors, publication year, study design, location and setting, period of investigation, study population) as well as the proportion of co‐infected patients and the pathogens implicated, method and time of detection of co‐infection, and characteristics describing the mono‐infected (SARS‐CoV‐2 only), and co‐infected subgroup (gender distribution, ICU‐admission‐rate, symptoms, case‐fatality‐rate).

### 2.3 Nomenclature and definitions

Terms for virus species and standard abbreviations are approved by the International Committee on Taxonomy of Viruses.^10^ Season division follows the meteorological periods (Northern Hemisphere: Winter [Dec‐Feb]; Spring [Mar‐May]; Summer [Jun‐Aug]; Autumn [Sep‐Nov], Southern Hemisphere: Winter [Jun‐Aug]; Spring [Sep‐Nov]; Summer [Dec‐Feb]; Autumn [Mar‐May]). Continents/geographic regions are classified according to the United Nations Standard Country or Area Codes for Statistical Use.^11^

### 2.4 Risk of bias assessment

The quality of included studies was examined using the Newcastle‐Ottawa Scale (NOS).^12^ The tool provides a maximum score of 4 for selection, 2 for comparability, and 3 for outcome. High‐quality studies have a score of >7 and moderate‐quality studies have a score of 5–7. Quality assessment was performed independently by two authors (HK, LK).

### 2.5 Outcomes

The main outcome we sought to analyse is the proportion of COVID‐19 patients who were co‐infected simultaneously with other respiratory viruses and to describe the co‐pathogens. Separate prevalence analyses were conducted for subgroups based on patients (gender, age) and study characteristics (location and time of investigation, cohort size of patients recruited). As secondary outcomes of interest, the rates of (i) respiratory tract infection symptoms (cough, fever, dyspnoea), (ii) intensive care unit admission, and (iii) fatality amongst co‐infected COVID‐19 patients were further assessed and compared to mono‐infected COVID‐19 patients. In addition to the co‐infection associated clinical outcomes, to evaluate the patients' gender as a potential risk factor for co‐infection, the rates of co‐infection among the male and female patients were compared.

### 2.6 Data analysis

Statistical analysis was carried out using the R (v4.1.2) package meta (v5.0‐0). Meta‐analysis of co‐infection prevalence was pooled by fitting a random intercept logistic regression model with the metaprop function to logit transformed proportions to include valid estimates for studies with very few or no co‐infections. Study estimates are shown with computed Clopper‐Pearson 95% confidence intervals. Heterogeneity was assessed by estimating the maximum‐likelihood of *τ* ^*2*^ and quantified with the *I* ^*2*^ index (*I* ^2^ > 75% indicates high heterogeneity^13^). Groupwise comparisons of studies with individual figures by gender and mono versus co‐infected with SARS‐CoV‐2 were analysed with the metabin function using a random effects model for the pooled Odds ratio (OR) with inverse variance weighting. Risk of publication bias (RoB) was assessed using a funnel plot of the logit transformed prevalence and inverse variance and tested using the metabias function with linear regression^14^ and rank correlation test^15^ for asymmetry. The quality of evidence (QoE) was evaluated using GRADE methods, which cover RoB, inconsistency, indirectness, imprecision, and publication bias.^13^ The QoE was evaluated for each outcome and described in the summary of findings tables, which were created with the GRADEpro GDT software.^16^

## 3 RESULTS

**FIGURE 1:**
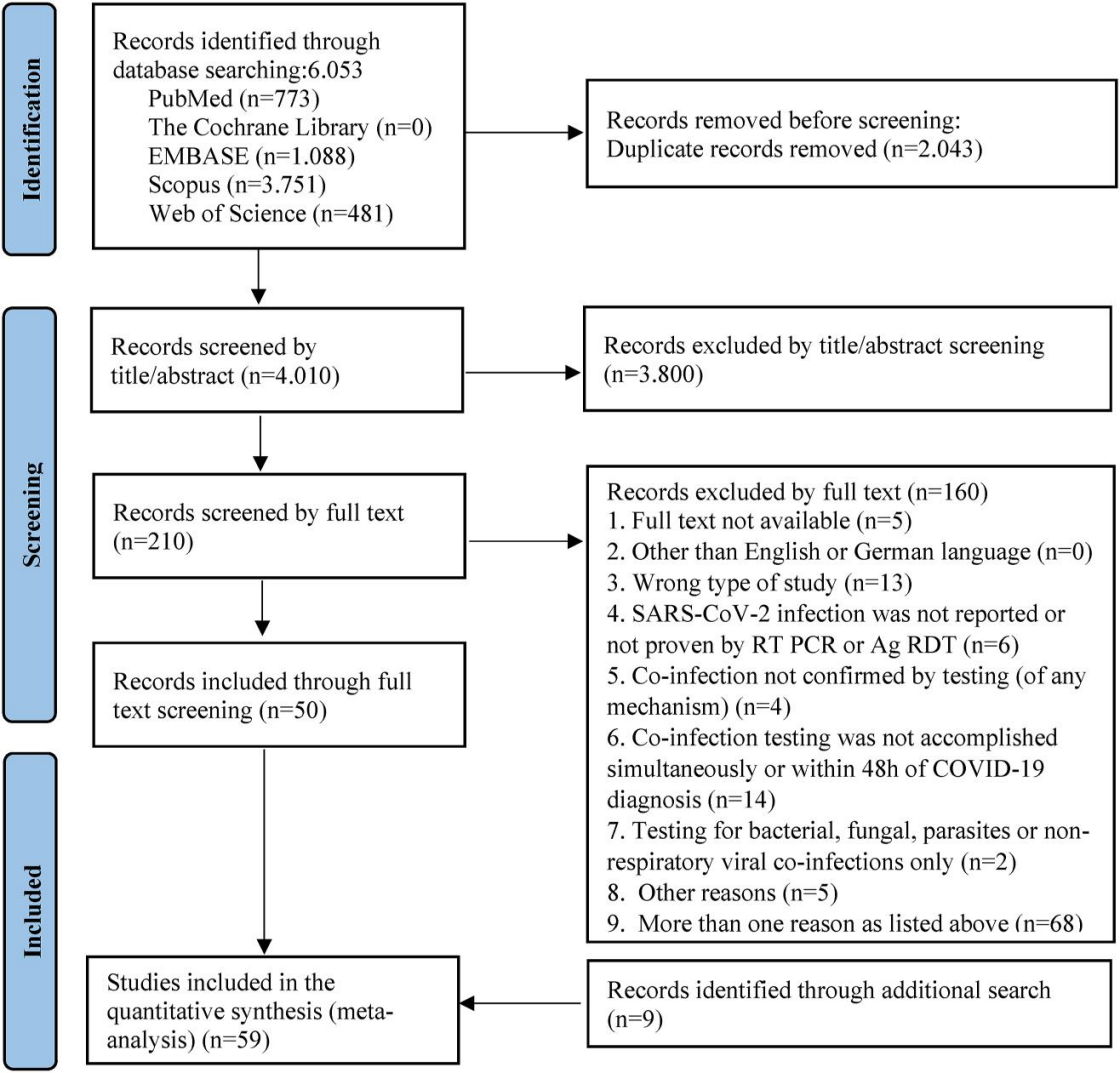
PRISMA^8^ flow diagram indicating the screening process to identify studies reporting on respiratory viral co‐infection rates in COVID‐19 patients. Abbreviations: SARS‐CoV‐2: severe acute respiratory syndrome‐related coronavirus 2; RT PCR: reverse‐transcription polymerase chain reaction (RT‐PCR) test; Ag RDT: antigen rapid diagnostic tests; COVID‐19: coronavirus disease 2019

We systematically searched PubMed, Web of Science, Embase, Scopus, and The Cochrane Library for studies published from 1 November 2019 up to 13 August 2021 (Figure 1). Of 6053 identified, 4010 records were screened and 3800 were found to be irrelevant based on their titles and abstracts. Of the remaining 210 records, 160 were excluded through more than one of the predefined exclusion criteria and the most frequent exclusion reason was a co‐infection test after 48 h of COVID‐19 diagnosis or missing information on the time of testing. 50 studies were included after full text screening. An additional manual search, by screening preprint servers and published meta‐analyses, provided nine records, which resulted in a total of 59 studies that were included in the meta‐analysis. A total sample size of 149,319 including 16,643 SARS‐CoV‐2 positive patients were analysed.

### 3.1 Characteristics of the included studies

Approximately two thirds of the studies were cohort studies (41/59). Forty‐three were single‐centred studies, whereas 16 studies were performed at multiple centres. Most of the studies were conducted in Asia (26/59), Europe (14/59), and North America (13/59). The majority of studies recruited patients without age restrictions (30/59). Twenty‐nine studies included adult patients and five studies paediatric patients only. While 50 of the studies reported a simultaneous laboratory screening for COVID‐19 and co‐infections, nine studies detected the co‐pathogen within 48 h of COVID‐19 diagnosis. To detect SARS‐CoV‐2 and co‐pathogens most of the studies (49/59) used a reverse‐transcription polymerase chain reaction (RT‐PCR) from nasopharyngeal and oropharyngeal swabs or tracheal aspirate. 10 studies applied either a metagenomic sequencing or immunofluorescence assay laboratory technique with or without an additional RT‐PCR assay. Since studies screened for different respiratory viruses, the number of COVID‐19 patients screened per virus type ranges from 166 patients that were screened for Middle East respiratory syndrome‐related coronavirus (MERS‐CoV) to 16,445 patients that were screened for Influenza Virus (FLU). The characteristics of included studies are summarised in Table S3.

The risk of bias was assessed by the NOS and is presented in Figure S1. The NOS score ranged from 6 to 9, with a median score of 7.3, which is indicative of moderate quality. Twenty‐two studies (37%) are rated as having high quality.

### 3.2 Overall pooled prevalence and subgroup analyses

**FIGURE 2:**
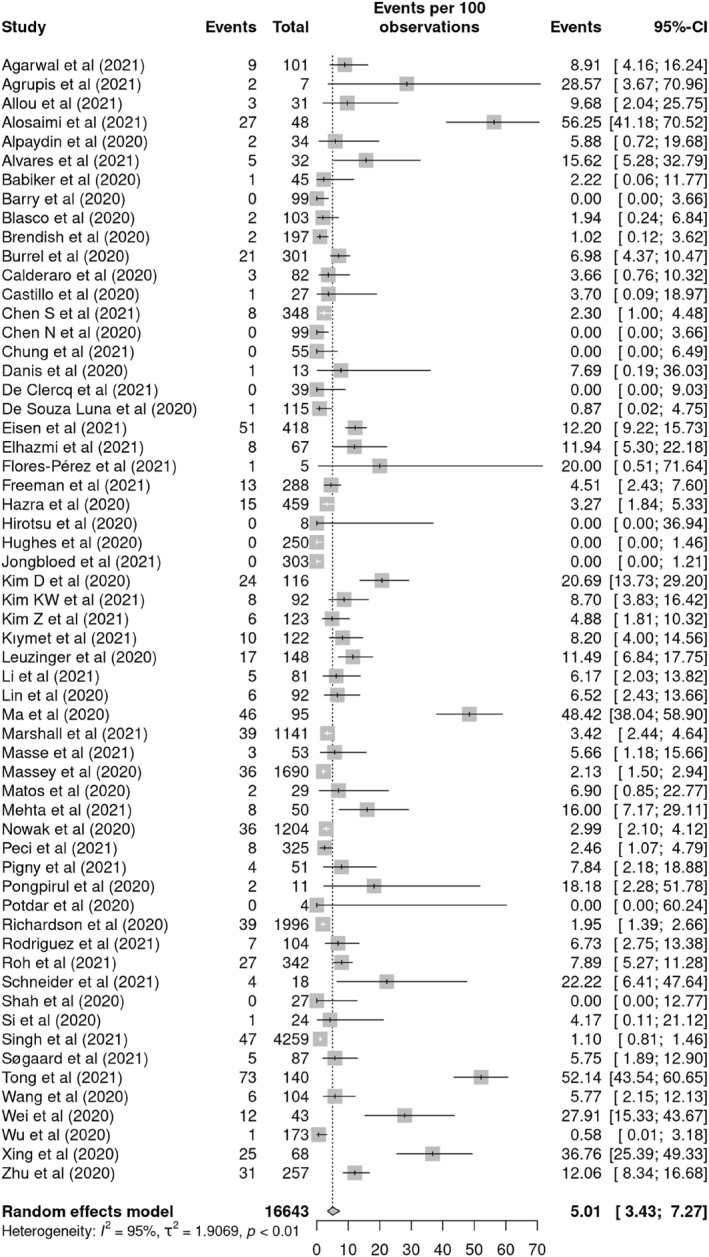
Forest plot of pooled prevalence of respiratory viral co‐infections (RVCI) among COVID‐19 patients according to the random effects approach. Each included study is presented by the first author and year of publication. Abbreviation: CI, confidence interval

By performing a random effects analysis, the pooled estimated prevalence of RVCI in COVID‐19 was 5.01% (95% CI 3.43%–7.27%, *n* = 16,643, 59 studies, *I* ^2^ = 95%) (Figure 2). The prevalence of co‐infection in the included studies ranged from 0%^17^, ^18^, ^19^, ^20^, ^21^, ^22^, ^23^, ^24^, ^25^ to 56.25%.^26^

**FIGURE 3:**
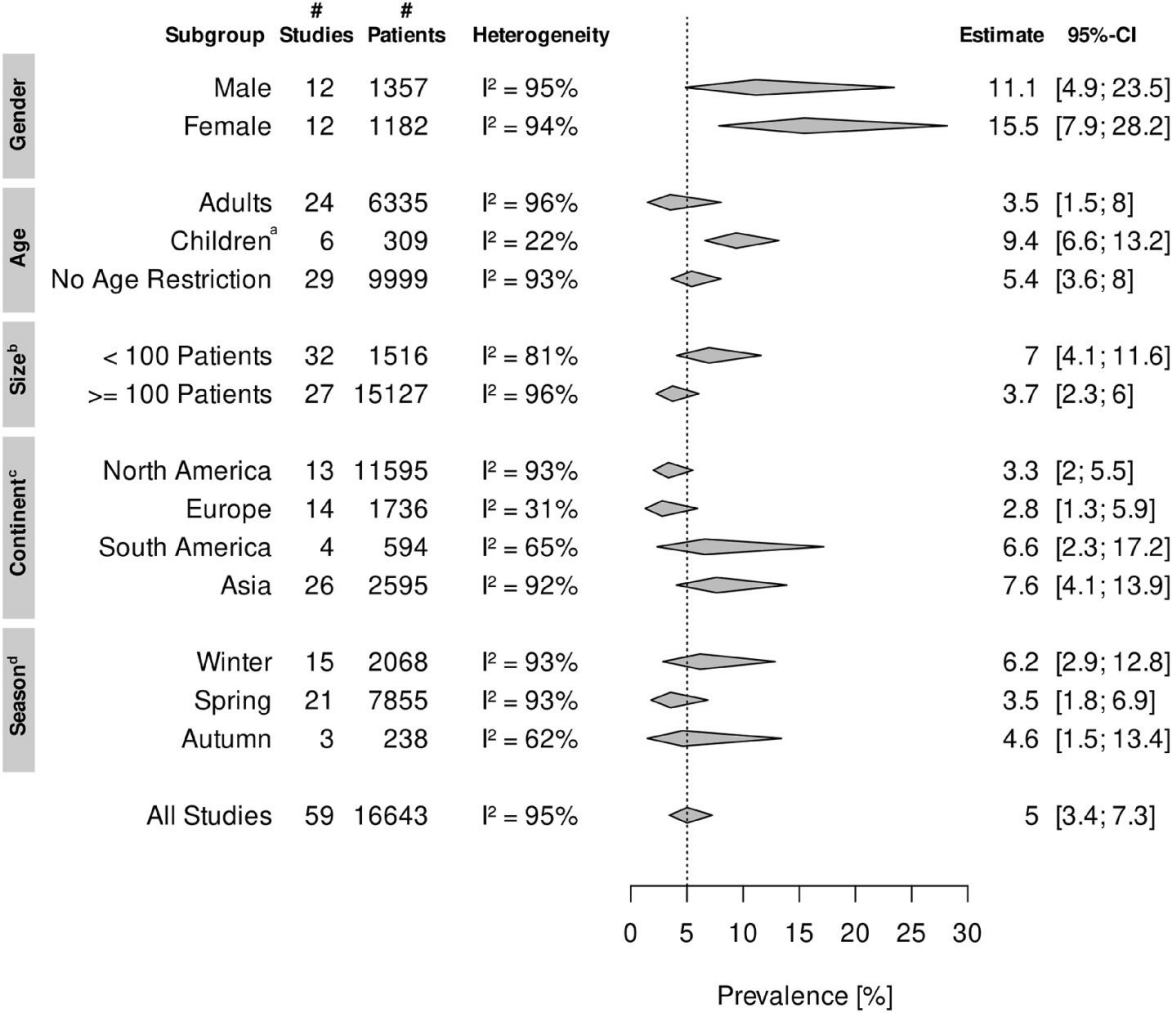
Meta‐analysis of respiratory viral co‐infection prevalence among COVID‐19 subgroups (Figure S2–S6). Abbreviations: CI, confidence interval; SARS‐CoV‐2, Severe acute respiratory syndrome‐related coronavirus. ^a^Patients under the age of 18 years. ^b^Size of SARS‐CoV‐2 positive patients tested for co‐infection. ^c^Continents Africa and Oceania are not demonstrated, since only one study was identified for each of them^27^, ^28^. ^d^Summer is not showed, since studies that screened COVID‐19 patients for co‐infections during the summer months overlap with the Spring or Autumn season. Studies from tropical geographic regions (*N* = 2^29^, ^30^) have been left out because they only experience a dry and a wet season.

Subgroup analysis between male and female COVID‐19 patients revealed that RVCI were more frequent in the female subgroup [15.45% (95% CI 7.85–28.17) and 11.11% (95% CI 4.85–23.45) in females and males respectively] (Figure 3). Analysis of studies with separate data for paediatric patients showed that RVCI is statistically significant more prevalent among children than adult patients (9.39% [95% CI 6.60–13.18], versus 3.51% [95% CI 1.49–8.04], respectively; *p*‐value = 0.02). The prevalence of RVCI was higher in Asia (7.36% [95% CI 4.06–13.87]) and South America (6.58% [95% CI 2.34–17.17]) than in Europe (2.78% [95% CI 1.28–5.94]) and North America (3.35% [95% CI 2.01–5.53]). Studies were assigned to different meteorological seasons based on the periods of investigation and study location. The highest prevalence was found during winter (6.17% [96% CI 2.85–12.84]), followed by autumn (4.60% [95% CI 1.48–13.40]) and spring (3.54% [95% CI 1.80–6.86]). In the subgroup of studies with less than 100 COVID‐19 patients a higher proportion of patients with RVCI was observed (7%) than in the one with studies with 100 and more COVID‐19 patients (3.7%). Only subgrouping by age showed a statistically significant difference in RVCI prevalence, while other subgroup analyses did not satisfy the required significance threshold.

### 3.3 Specific prevalence of respiratory co‐pathogens

**FIGURE 4:**
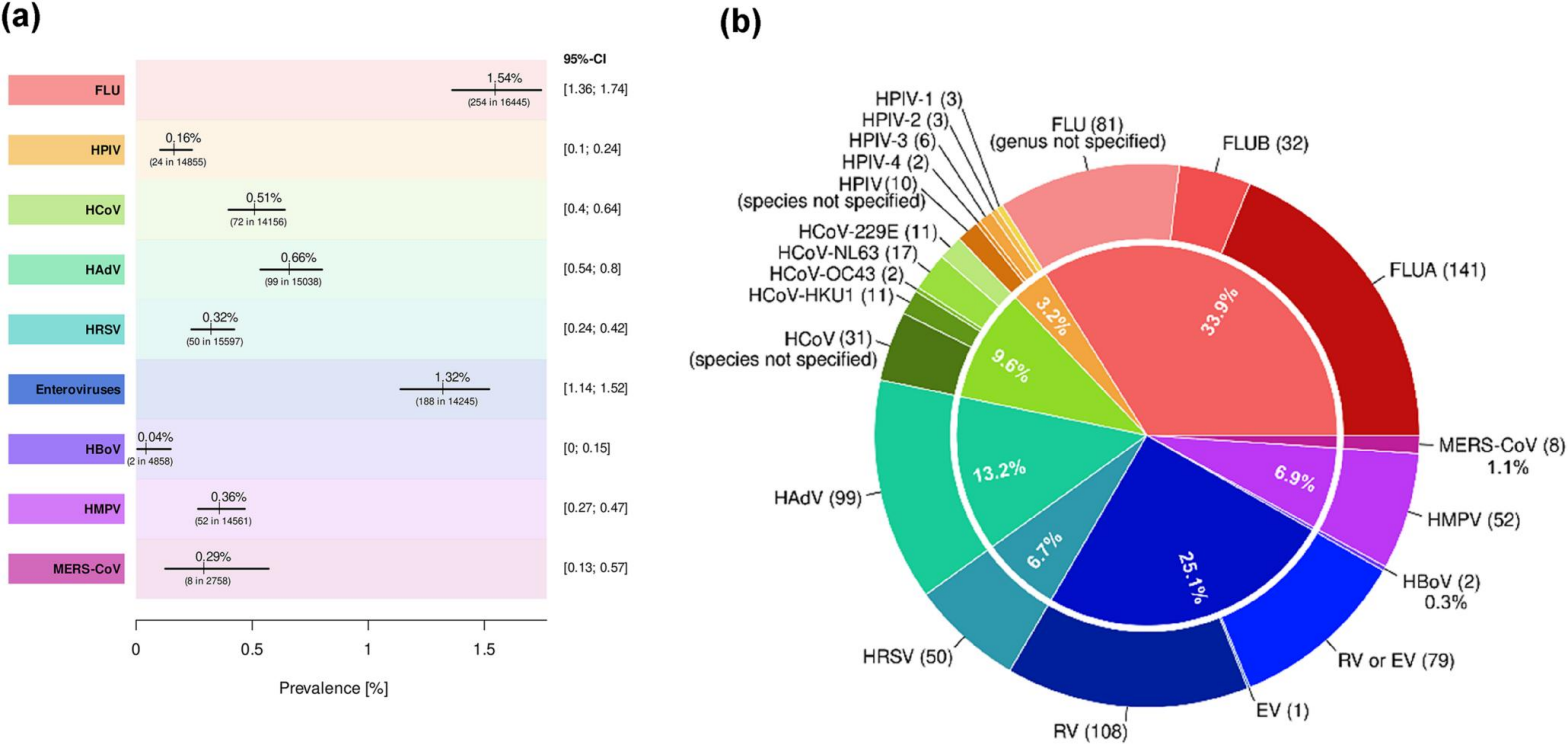
Prevalence and proportion of each respiratory virus identified in COVID‐19 patients. (a), Pooled prevalence of each respiratory virus identified in co‐infected COVID‐19 patients based on the number of tests performed for each pathogen. Each line segment's midpoint exhibited the prevalence estimation; the line segment length presents 95% confidence intervals. (b), Number of respiratory viral pathogens detected in COVID‐19 patients, as a proportion of the total number of detections (*N* = 749). Abbreviations: FLU, Human influenza virus; FLUA, Human influenza virus A; FLUB, Human influenza virus B; HPIV, Human parainfluenza virus; HPIV‐1, Human parainfluenza virus 1; HPIV‐2, Human parainfluenza virus 2; HPIV‐3, Human parainfluenza virus 3; HPIV‐4, Human parainfluenza virus 4; HCoV, Human respiratory coronavirus; HCoV‐229E, Human respiratory coronavirus 229E strain; HCoV‐NL63, Human respiratory coronavirus NL63 strain; HCoV‐OC43, Human respiratory coronavirus OC43 strain; HCoV‐HKU1, Human respiratory coronavirus HKU1 strain; HAdV, Human adenovirus; HRSV, Human respiratory syncytial virus; RV, Human rhinovirus; EV, Human enterovirus; HBoV, Human bocavirus; HMPV, Human metapneumovirus; MERS‐CoV, Middle East respiratory syndrome‐related coronavirus; HPeV, Human parechovirus

A total of 749 respiratory viral co‐pathogens were identified in 52/59 studies (Figure 4). FLU (*N* = 254, 33.92% of detections) and Enteroviruses (*N* = 188, 25.10% of detections) were the most prevalent pathogens; with the FLUA (*N* = 141/254) and human rhinoviruses (RV) (*N* = 108/188) representing the majority of each genus respectively, followed by 99 human adenovirus (HAdV) and 72 common human coronavirus (HCoV) isolates. The minority of viruses observed among the included studies were: human parainfluenza virus (HPIV), MERS‐CoV, and human bocavirus (HBoV). Considering the tests conducted per each virus, the proportions correlate with the specific prevalences of each co‐pathogen. With the largest number of tests performed in 56/59 studies, FLU reaches the highest level of co‐infection prevalence (1.54%). Enteroviruses (1.32%), which were screened in 18/59 studies are the second most prevalent respiratory co‐viruses. For HAdV (0.66%), HCoV (0.51%), human metapneumovirus (0.36%), human respiratory syncytial virus (HRSV) (0.32%), MERS‐CoV (0.29%), HPIV (0.16%), HBoV (0.04%) less than 10 out of 1000 tests were positive.

### 3.4 Comparison of SARS‐CoV‐2 mono‐ and co‐infected patients

The impact of RVCI on the patients' outcome was investigated by comparison of the mono‐infected (SARS‐CoV‐2 only) and co‐infected COVID‐19 patients (Table 1). While there were no major differences in the occurrence of cough and fever, co‐infected COVID‐19 patients were more likely to suffer from dyspnoea than SARS‐CoV‐2 mono‐infected patients (48.1% vs. 37.3% of the patients, respectively). Almost equal rates of patients needed to be submitted to an ICU (25.6% vs. 25.3% of the patients), however, the case‐fatality‐rate was almost three times higher in the co‐infected than mono‐infected subgroup (18.2% vs. 6.7% of the patients). Patients' gender as a risk factor for RVCI was assessed by exploring the OR of co‐infection among the male and female subgroup, which revealed no contributable effect. None of the other secondary outcomes resulted in an OR significantly different from 1.

**TABLE 1:** Comparison of the mono‐infected (SARS‐CoV‐2 only) and co‐infected (SARS‐CoV‐2 and one or more respiratory viruses) patient groups by secondary outcome measurements (Figure S7–S10)

Secondary outcome	No. of studies	No. of patients (Events/Total)		*p‐v*alue	Relative effect (95% CI)	I‐squared
		SARS‐CoV‐2 co‐infected patients	SARS‐CoV‐2 mono‐infected patients			
Symptom cough	5	82/131 (62.6%)	136/225 (60.4%)	0.21	OR 0.72 (0.42–1.12)	8%
Symptom fever	5	95/131 (73.3%)	171/225 (76.0%)	0.12	OR 0.63 (0.35–1.12)	28%
Symptom dyspnoea	5	63/131 (48.1%)	84/225 (37.3%)	0.94	OR 0.97 (0.38–2.47)	41%
ICU‐admission‐rate	7	20/78 (25.6%)	228/901 (25.3%)	0.75	OR 0.89 (0.42–1.87)	0%
Case‐fatality‐rate	10	37/203 (18.2%)	42/626 (6.7%)	0.48	OR 1.66 (0.40–6.78)	67%
Gender distribution		Co‐infections among males	Co‐infections among females			
	12	136/1357 (10.0%)	133/1182 (11.3%)	0.12	OR 0.79 (0.59–1.06)	0%

Abbreviations: CI, confidence interval; ICU, intensive care unit; OR, Odds ratio; SARS‐CoV‐2, Severe acute respiratory syndrome‐related coronavirus.

### 3.5 Publication bias and quality of evidence assessment

Publication bias was evaluated using funnel plots (Figure S11). Begg's correlation test (*z* = −1.26, *p*‐value = 0.2092) and Egger regression (*t* = −0.28, *p*‐value = 0.7820) revealed no publication bias. Based on GRADE,^16^ the overall QoE across all studies was low (Table S4 and S5).

## 4 DISCUSSION

In this extensive systematic review and meta‐analysis, we found a prevalence of 5% of RVCI among COVID‐19 patients. FLUA, RV, and HAdV were the most common identified co‐pathogens (18.83%, 14.42% and 13.22% of all detections, respectively). Subgroup analyses showed that co‐infection was significantly higher in paediatric (9.39%) than adult (3.51%) patients (*p*‐value = 0.02). Furthermore, co‐infected patients were more likely to be dyspnoeic and the odds of fatality (OR = 1.66) were increased.

To our knowledge, this is the first study that explicitly focussed on respiratory viruses as co‐pathogens in COVID‐19 patients. Additionally, we included only concurrent community‐acquired and not secondary or hospital‐acquired infections by excluding studies that tested for co‐infections after 48 h of COVID‐19 hospitalisation.

The prevalence RVCI in COVID‐19 patients is difficult to estimate accurately. Respiratory viruses, including SARS‐CoV‐2, often cause similar clinical features; this, along with the fact that not all laboratories have the same capacity, and economic and human resources to set up multiplex testing techniques, especially during a pandemic, are the major limitations in distinguishing viruses from each other.^31^

Previous reviews found RVCI rates of 12.58%^32^ and 10%, with FLUA, FLUB and HRSV^33^ being the most prevalent, in COVID‐19 patients. These meta‐analyses either included studies, which tested for all types of viruses or did not state the tested viruses, which might be the reason for the higher co‐infection rates identified. During the H1N1 influenza virus pandemic in 2009, higher rates of RVCI were observed (13.1%^34^), which can be explained by the lower infection control measures in contrast to the COVID‐19 pandemic. RV were the most frequently identified co‐infecting pathogens in H1N1 influenza patients. Comparing SARS‐CoV‐2 with other members of the betacoronavirus genus, MERS‐CoV has a higher rate of respiratory viral co‐infections (10.3%)^35^ and there is evidence of a much lower co‐infection rate (0.01%) for SARS‐CoV‐1.^36^

Our subgroup analyses revealed that the rate of co‐infection was significantly higher in paediatric than in adult patients (9.39% vs. 3.51%, *p*‐value = 0.03). These results are consistent with a recent study examining co‐infection rates for paediatric (10.0%) and adult (2.4%) patients.^37^ The immature immune system in the youngest patients compared to adults and the greater interaction of children can explain the higher probability of co‐infection detection. Although meta‐analysis revealed a small increase in the co‐infection rate for female patients (11.1% in male vs. 15.5% in female, *p*‐value = 0.12), the difference did not reach statistical significance. In the subgroup containing studies with less than 100 COVID‐19 patients the prevalence was higher (from 5.01% to 7%). Underpowered studies often contribute little information and possibly cause risk of bias. It is therefore advisable that future reviews should include studies with a higher minimum number of COVID‐19 patients tested for co‐infections.^38^ The prevalences of RVCI differ greatly among continents. This can be attributed to the low number of studies per subgroup, as well as to the different international laboratory infrastructures and temporal course of the pandemic depending on the geographic region.

Our study showed that co‐infection was associated with a higher case‐fatality rate (6.7% in mono‐infected vs. 18.2% in co‐infected patients), which is consistent with other studies showing a positive association between co‐infection and increased risk of death among co‐infected COVID‐19 patients.^33^ Interestingly, while co‐infected patients were more likely to be dyspnoeic, fever and cough occurred at similar rates in the mono‐ and co‐infected patients.

### 4.1 Strength and limitation

There are three major strengths of our study; firstly, we distinguished between community and hospital‐acquired infections by strictly selecting studies that screened for co‐infections within 48 h of COVID‐19 hospitalisation. Secondly, we recorded the co‐viruses identified in the included studies, which allowed us to specify their individual prevalence. Thirdly, the periods of investigation of our studies reach from 1 20 December19^39^ till 11 20 March21^40^ and are evenly distributed throughout the years. Seasonal activity of the respiratory viruses can influence the prevalence of co‐infections. In our meta‐analysis 32 studies enroled patients mainly during the colder months from December to March and 27 studies represent ‘no‐Flu’ months and locations, which do not experience typical seasons.

Some limitations should be acknowledged in this review. Firstly, neither the included microbiological testing methods nor the viral pathogens tested are uniform across studies. This led to a huge disparity in the number of tests performed per virus. This may affect the observed prevalence. Secondly, we included all types of patients without restrictions to setting, disease severity and possible co‐morbidities. Most patients screened were symptomatic. There is a paucity of data regarding the rate of co‐infections in mildly symptomatic or asymptomatic COVID‐19 patients. Furthermore, the effect of co‐morbidities on the prevalence and outcome of RVCI could not be assessed. Thirdly, the underrepresentation of data from Africa, Australia and South America due to the lack of studies may distort the actual global prevalence, while it reduces the statistical power of subgroup analysis for continents. Finally, the analysis was limited to English and German literature and thus may miss studies published in other languages.

### 4.2 Conclusion

In conclusion, the present systematic review and meta‐analysis provides evidence of an overall co‐infection prevalence of 5.01% among COVID‐19 patients. Although the incidence of COVID‐19 remains high, the prevalence of respiratory viral co‐infections is relatively low. The worldwide infection control measures may have played a role in reducing the circulation of respiratory viruses.

Our findings show that multiplex viral panel testing may be advisable in patients with compatible symptoms. Particularly for FLU, assessment for co‐infection could have major prognostic and treatment implications. Further studies are required to independently confirm our observations. To clearly establish the importance of detecting RVCI among COVID‐19 patients, it is necessary to investigate the patients' outcome for each co‐virus separately and differentiate between children and adults.

## AUTHOR CONTRIBUTIONS

Hanna Krumbein and Lara S. Kümmel contributed equally to this study. Hanna Krumbein, Chrysanthi Skevaki and Paraskevi C. Fragkou conceptualised the study. Hanna Krumbein and Lara S. Kümmel performed the literature search and Hanna Krumbein, Lara S. Kümmel, Ben L. Hünerbein and Rieke Reiter performed the study screening and selection. Hanna Krumbein and Lara S. Kümmel extracted and verified the study data. Clemens Thölken did the analysis. Hanna Krumbein, Chrysanthi Skevaki and Paraskevi C. Fragkou wrote the first draft of the manuscript. All authors had access to all data in the study and have read and approved the published version of the manuscript.

## CONFLICTS OF INTEREST

For Chrysanthi Skevaki: Consultancy and research funding, Hycor Biomedical, Bencard Allergie and Thermo Fisher Scientific; Research Funding, Mead Johnson Nutrition (MJN).

## Supporting information

Supporting Information S1

## Data Availability

The authors confirm that the data supporting the findings of thisstudy are available within the article and its supplementary materials.
