# Supplementary material for: Respiratory viral co‐infections in patients with COVID‐19 and associated outcomes: A systematic review and meta‐analysis
Source: Rev Med Virol. 2022 Jun 10:e2365. Online ahead of print. doi: 10.1002/rmv.2365 (PMC9347814; doi:10.1002/rmv.2365)
Supplement: Supplementary file 1 — Supporting Information S1 [file RMV-9999-0-s001.docx]

**Supplementary Material**

Respiratory viral co-infections in patients with COVID-19 and associated outcomes: A systematic review and meta-analysis

1. Supplementary Tables
   1. **Supplementary Table S1.** Search concepts and search strings
   2. **Supplementary Table S2.** PICOS algorithm
   3. **Supplementary Table S3.** Summary of the characteristics of the included studies with evidence on SARS-CoV-2 and respiratory viral co-infections (N=59)
   4. **Supplementary Table S4.** GRADE Evidence Profile
   5. **Supplementary Table S5.** Summary of Findings of Secondary Outcome Sub-analyses
   6. **Supplementary Table S6.** PRISMA Checklist
2. Supplementary Figures
   1. **Supplementary Figure S1.** Summary of Newcastle-Ottawa Scale Quality Assessment
   2. Forrest plots of pooled prevalence in COVID-19 patients’ subgroups
      1. **Supplementary Figure S2.** Pooled prevalence of co-infection rates among the female (A) and male (B) COVID-19 patients’ subgroups.
      2. **Supplementary Figure S3.** Pooled prevalence of co-infection rates among studies including pediatric (A) or adult (B) patients only and those with no age restriction (C).
      3. **Supplementary Figure S4.** Pooled prevalence of co-infection rates in studies that screened < 100 SARS-CoV-2 positive patients (A) and studies that screened ≥ 100 SARS-CoV-2 positive patients (B).
      4. **Supplementary Figure S5.** Pooled prevalence of co-infection rates among the different continents/geographic regions Asia (A), North America (B), Europe (C) and South America (D).
      5. **Supplementary Figure S6.** Pooled prevalence of co-infection rates for Spring (A), Autumn (B) or Winter (C) season.
   3. Subgroup Analyses of Secondary Outcomes
      1. **Supplementary Figure S7.** Odds ratios of co-infection in male vs. female COVID-19 patients.
      2. **Supplementary Figure S8.** Odds ratio of the ICU-Admission-Rate in the co-infected vs. mono-infected patient groups.
      3. **Supplementary Figure S9.** Odds ratio of the Case-Fatality-Rate in the co-infected vs. mono-infected patient groups.
      4. **Supplementary Figure S10.** Odds ratio of the presence of cough (A), fever (B) and dyspnea (C) in the co-infected vs. mono-infected patient group.
   4. **Supplementary Figure S11.** Funnel plot on the reported co-infection rates among COVID-19 patients.
3. References

**Supplementary Table S1.** Search concepts and search strings

| **Concept** | **Search strings** |
| --- | --- |
| 1) COVID-19 | “covid 19” OR “covid-19” OR “covid19” OR “covid*” OR “SARS-CoV-2” OR “SARS CoV 2” OR “SARS coronavirus 2” OR “SARS-CoV-2 infection*” OR “2019 nCoV” OR “nCoV*” OR "Severe Acute Respiratory Syndrome Coronavirus 2" OR “coronavirus infection” OR “coronavirus disease 19” OR “covid-19 virus disease*” |
| 2) Co-infection | "co-infect*" OR "co infect*" OR "coinfect*" OR "concurrent infection" OR "concurrent infections" OR "concomitant infection" OR "concomitant infections" OR "mixed infection" OR "dual infection" |
| 3) Other than SARS-CoV-2 respiratory viruses | “viral” OR “virus*” OR “viral pathogen” OR "respiratory syncytial viruses" OR "respiratory syncytial virus*" OR "RSV" OR "metapneumovirus" OR "metapneumovir*" OR "meta pneumovir*" OR "HMPV" OR "influenza" OR "influenzavirus A" OR "influenzavirus B" OR "influenza virus C" OR "influenza*" OR "seasonal flu" OR "grippe" OR "flu" OR "MERS" OR “SARS-CoV-1” OR “SARS CoV 1” OR "alphacoronavirus" OR "betacoronavirus" OR "229E" OR "NL63" OR "OC43" OR "HKU1" OR "parainfluenza" OR "parainfluenza virus" OR "adenovirus" OR "adenoviridae" OR "bocavirus" OR "human bocavirus" OR "HBoV" OR "rhinovirus" OR "rhino virus" OR "enterovirus" OR "entero virus" |
| 4) Publication Date | 01/11/2019-13/08/2021 |
| 5) Language | English OR German |
| All terms within each concept combined with OR, all concepts with AND. | |

**Supplementary Table S2.** PICOS algorithm

|  | **INCLUSION CRITERIA** | **EXLCUSION CRITERIA** |
| --- | --- | --- |
| **Patients/ Participants** | ●adult and paediatric patients (no age restriction)  ●inpatients and outpatients  ●COVID-19 of any severity  ●confirmed Sars-CoV-2 cases, according to WHO COVID-19 case definition (proven by Ag-RDT (antigen rapid detection test) or Nucleic Acid Amplification Test, like RT-PCR test)^1^ | ●cases not fulfilling WHO definition of COVID-19 |
| **Intervention** | ●Test for community-acquired (for admitted patients identified within 48 h of hospitalization^2^) co-infection with respiratory viruses proven (any test mechanism (PCR, serology, e.g.))  respiratory viruses:  human influenza viruses (A, B), human parainfluenza virus (1, 2, 3, 4), human coronavirus (229E, NL63, OC43, HKU1), human adenoviruses (HAdV), human respiratory syncytial virus (HRSV), enteroviruses (EV, RV), human bocavirus (HBoV), human metapneumovirus (HMPV), Middle East respiratory syndrome-related coronavirus (MERS-CoV), human parechovirus (HPeV) | ●co-infection with bacteria, fungi or non-respiratory viruses only  non-respiratory viruses:  herpes simplex viruses (HSV-1 and HSV-2), Epstein-Barr virus (EBV / HHV-4), human herpesvirus 6 (HHV 6), human cytomegalovirus (HCMV), human immunodeficiency viruses (HIV), Dengue virus (DENV), Zika virus (ZIKV), human papillomavirus (HPV), hepatitis viruses (A, B, C, D, E), rhabdovirus, Torque teno virus (TTV), varicella zoster virus (VZV / HHV-3) |
| **Comparison/ Comparator** | No restrictions on comparator, studies without interventions. | NA |
| **Outcomes** | Main Outcome:  ●Prevalence of pathogens in respiratory co-infections with viruses other than SARS CoV-2 (any testing mechanism)  Additional Outcomes:  ● Differences in clinical features between mono-infected (only Sars-CoV-2) and co-infected patients (e.g., age distribution, gender distribution, geographical distribution, symptoms) | NA |
| **Studies** | ●Randomized controlled trials, prospective and retrospective cohort, cross-sectional, case-control or observational studies, case series ≥ 10 participants  ●English and German language | ●Reviews, conference abstracts, case series < 10 participants, case reports, epidemiological registry data, animal studies  ●Full text not available |

NA: not available

**Supplementary Table S3.** Summary of the characteristics of the included studies with evidence on SARS-CoV-2 and respiratory viral co-infections (N=59)

| **Study** | **Study Location** | **Study type** | **Population** | **Period of investigation** | **Number of SARS-CoV-2 Patients Tested for Co-Infection** | **Number of Co-Infected Patients, n (%)** | **Laboratory Techniques for Co-Pathogen Detection**  **(Testing Panel)** | **Type of Respiratory Viruses (other than SARS-CoV-2) tested** | **Number of detected respiratory viruses and identiy** | **Quality of Study** |
| --- | --- | --- | --- | --- | --- | --- | --- | --- | --- | --- |
| Agarwal  et al (2021) ^3^ | India | Singlecenter, retrospective cohort study | COVID-19 confirmed  admitted  patients | 01/08/2020 to 31/12/2020 | 101 | 9 (8.9) | RT-PCR from naso- or oropharyngeal swab | FLUA, FLUB | *9 FLUA* | high |
| Agrupis  et al (2021) ^4^ | Philippines | Singlecenter, retrospective cohort study | Health Care Workers | 20/03/2020 to 20/04/2020 | 7 | 2 (28.6) | multiplex RT-PCR from naso- or oropharyngeal swab | FLUA, FLUB, HPIV-1, HPIV-2, HPIV-3, HPIV-4, HCoV-229E, HCoV-NL63, HCoV-OC43, HCoV-HKU1, HAdV, HRSV, RV, HBoV, HMPV | *2 FLUA*  *1 HPIV-1* | moderate |
| Allou  et al (2021) ^5^ | France (Réunion) | Singlecenter, prospective cohort study | COVID-19 confirmed  adult patients | 18/03/2020 to 15/04/2020 | 31 | 3 (9.7) | multiplex RT-PCR from nasopharyngeal swab in non-intubated and tracheal aspirate in intubated patients  (Seegene Allplex respiratory panel) | FLUA, FLUB, HPIV-1, HPIV-2, HPIV-3, HPIV-4, HCoV-229E, HCoV-NL63, HCoV-OC43, HCoV-HKU1, HAdV, HRSV, RV, EV, HBoV, HMPV | *1 IVA*  *1 HCoV-229E*  *1 RV* | moderate |
| Alosaimi  et al (2021) ^6^ | Saudi Arabia | Singlecenter, retrospective cohort study | COVID-19 confirmed  admitted patients | 27/04/2020 to 27/05/2020 | 48 | 27 (56.3) | multiplex RT-PCR from nasopharyngeal swab (FTD Respiratory Pathogens 21) | FLUA, FLUB, HPIV-1, HPIV-2, HPIV-3, HPIV-4, HCoV-229E, HCoV-NL63, HCoV-OC43, HCoV-HKU1, HAdV, HRSV, RV, EV, HBoV, HMPV, HPeV | *17 FLUA*  *1 FLUB*  *1 HPIV-3*  *10 HAdV*  *1 HMPV* | high |
| Alpaydin  et al (2020) ^7^ | Turkey | Singlecenter, retrospective cross-sectional study | COVID‐19 suspected  admitted adult patients | 18/03/2020 to 31/03/2020 | 34 | 2 (5.9) | multiplex RT-PCR from nasopharyngeal swab (FTD Respiratory Pathogens 21) | FLUA, FLUB, HPIV-1, HPIV-2, HPIV-3, HPIV-4, HCoV-229E, HCoV-NL63, HCoV-OC43, HCoV-HKU1, HAdV, HRSV, RV, EV, HBoV, HMPV, HPeV | *1 HCoV-NL63*  *1 HAdV* | moderate |
| Alvares  et al (2021) ^8^ | Brazil | Singlecenter, retrospective cohort study | COVID-19 confirmed admitted children  under 24 months of age | 01/03/2020 to 30/09/2020 | 32 | 5 (15.6) | RT-PCR from  nasopharyngeal secretion | HRSV | *5 HRSV* | high |
| Babiker  et al (2020) ^9^ | United States of America | Singlecenter, retrospective analysis | COVID-19 suspected patients | 26/02/2020 to 23/04/2020 | 45 | 1 (2.2) | Metagenomic Sequencing, Flu/RSV PCR, BioFire® FilmArray® RP 2.1 Panel, BioFire® FilmArray® Pneumonia Panel | FLUA, FLUB, HPIV-1, HPIV-2, HPIV-3, HPIV-4, HCoV-229E, HCoV-NL63, HCoV-OC43, HCoV-HKU1, HAdV, HRSV, RV, EV, HMPV, MERS-CoV | *1 HRSV* | high |
| Barry  et al (2020) ^10^ | Saudi Arabia | Singlecenter, retrospective case series | COVID-19 confirmed admitted adult patients | 22/03/2020 to 31/05/2020 | 99 | 0 (0.0) | RT-PCR from nasopharyngeal or throat swab | MERS-CoV | *NA* | moderate |
| Blasco  et al (2020) ^11^ | Spain | Singlecenter, prospective cohort study | COVID -19 confirmed  admitted  adult patients | 04/03/2020 to 28/03/2020 | 103 | 2 (1.9) | multiplex PCR assay from naso- or oropharyngeal swab (NxTAG Respiratory Pathogen Panel) | FLUA, FLUB, HPIV-1, HPIV-2, HPIV-3, HPIV-4, HCoV-229E, HCoV-NL63, HCoV-OC43, HCoV-HKU1, HAdV, HRSV, RV or EV, HBoV, HMPV | *1 FLUA*  *1 HCoV-HKU1*  *1HRSV* | moderate |
| Brendish  et al (2020) ^12^ | United Kingdom | Multicenter, prospective cohort study | COVID-19 suspected adult patients | 20/03/2020 to 29/04/2020 | 197 | 2 (1.0) | multiplex RT-PCR (QIAstat-Dx Respiratory SARS-CoV-2 Panel) | FLUA, FLUB, HPIV-1, HPIV-2, HPIV-3, HPIV-4, HCoV-229E, HCoV-NL63, HCoV-OC43, HCoV-HKU1, HAdV, HRSV, RV, EV, HBoV, HMPV | *1 HCoV-HKU1*  *1 HAdV* | moderate |
| Burrel  et al (2020) ^13^ | France | Singlecenter, retrospective cohort study | COVID-19 suspected patients | 25/01/2020 to 29/03/2020 | 301 | 21 (7.0) | (Filmarray® RP2plus) | FLU, HPIV, HCoV, HAdV, HRSV, RV or EV, HMPV, MERS-CoV | *5 FLU (genus not specified)*  *2 HPIV (species not specified)*  *6 HCoV (species not specified)*  *4 HAdV*  *4 RV or EV* | high |
| Calderaro  et al (2020) ^14^ | Italy | Singlecenter, prospective analysis | patients with respiratory symptoms | 01/12/2019 to 31/03/2020 | 82 | 3 (3.7) | RT-PCR from nasopharyngeal aspirate, nasal or throat swab or sputum (Allplex Respiratory Panels 1–3), Immunofluorescence assay for RSV antigen | FLUA, FLUB, HPIV-1, HPIV-2, HPIV-3, HPIV-4, HCoV-229E, HCoV-NL63, HCoV-OC43, HAdV, HRSV, RV, EV, HBoV, HMPV | *1 HCoV-NL63*  *2 RV* | high |
| Castillo  et al (2020) ^15^ | United States of America | Multicenter, retrospective analysis | COVID-19 suspected patients | 10/03/2020 to 23/03/2020 | 27 | 1 (3.7) | RT-PCR from nasal or throat-swab (GenMark ePlex respiratory pathogens panel) | FLUA, FLUB, HPIV-1, HPIV-2, HPIV-3, HPIV-4, HCoV-229E, HCoV-NL63, HCoV-OC43, HCoV-HKU1, HAdV, HRSV, RV or EV, HMPV | *1 FLUA* | moderate |
| Chen S  et al (2021) ^16^ | China | Singlecenter, retrospective cohort study | COVID-19 confirmed  adult patients | 11/01/2020 to 01/03/2020 | 348 | 8 (2.3) | RT-PCR from nasopharyngeal swab (Cepheid Xpert® Xpress Flu/RSV) | FLUA, FLUB, HRSV | *1 FLUA*  *3 FLUB*  *4 HRSV* | high |
| Chen N  et al (2020) ^17^ | China | Singlecenter, retrospective cohort study | COVID-19 confirmed  patients | 01/01/2020 to 20/01/2020 | 99 | 0 (0.0) | RT-PCR from throat-swab | FLUA, FLUB, HPIV-1, HPIV-2, HPIV-3, HPIV-4, HAdV, HRSV, MERS-CoV | *NA* | moderate |
| Chung  et al (2021) ^18^ | Taiwan | Singlecenter, retrospective experimental study | COVID-19 suspected  patients | 01/02/2020 to 31/08/2020 | 55 | 0 (0.0) | dual multiplex PCR from nasopharyngeal swab | FLUA, FLUB, HRSV | *NA* | moderate |
| Danis  et al (2020) ^19^ | France | Singlecenter, retrospective case series | tourists that stayed in a chalet and their contact persons | 01/01/2020 to 29/02/2020 | 13 | 1 (7.7) | multiplex  RT-PCR from nasopharyngeal swab or endotracheal aspirates | FLUA, FLUB, HPIV-1, HPIV-2, HPIV-3, HPIV-4, HCoV-229E, HCoV-NL63, HCoV-OC43, HCoV-HKU1, HAdV, RV | *1 FLUA*  *1 RV* | moderate |
| De Clercq  et al (2021) ^20^ | Belgium | Singlecenter, retrospective cohort study | COVID-19 suspected  admitted patients | 19/03/2020 to 30/04/2020 | 39 | 0 (0.0) | RT-PCR from nasopharyngeal swab and BAL fluid (Seegene Allplex™ Respiratory Panel) | FLUA, FLUB, HPIV-1, HPIV-2, HPIV-3, HPIV-4, HCoV-229E, HCoV-NL63, HCoV-OC43, HCoV-HKU1, HAdV, HRSV, RV, EV, HBoV, HMPV | *NA* | moderate |
| De Souza Luna  et al (2020) ^21^ | Brazil | Singlecenter, retrospective cohort study | admitted patients with acute respiratory illness | 12/03/2020 to 16/04/2020 | 115 | 1 (0.9) | RT-PCR from nasal- or oropharyngeal swab | FLUA, FLUB, HRSV, HMPV | *1 FLUB* | moderate |
| Eisen  et al (2021) ^22^ | Brazil | Multicenter, retrospective analysis | COVID-19 suspected  admitted patients | 01/03/2020 to 31/12/2020 | 418 | 51 (12.2) | RT-PCR from naso‐ or oropharyngeal swabs or bronchoalveolar lavage | FLUA, FLUB, HAdV, RV, EV | *6 FLUA*  *20 HAdV*  *27 RV* | moderate |
| Elhazmi  et al (2021) ^23^ | Saudi Arabia | Singlecenter, retrospective case series | COVID-19 confirmed patients  admitted to ICU | 14/03/2020 to 19/10/2020 | 67 | 8 (11.9) | RT-PCR from nasopharyngeal swabs | MERS-CoV | *8 MERS-CoV* | moderate |
| Flores-Pérez  et al (2021) ^24^ | Spain | Multicenter, prospective cohort study | infants under 1 year of age, hospitalized with a diagnosis of acute bronchiolitis | 01/10/2020 to 31/12/2020 | 5 | 1 (20) | RT-PCR from nasopharyngeal swab  (FTD Respiratory Pathogens 21) | FLUA, FLUB, HPIV-1, HPIV-2, HPIV-3, HPIV-4, HCoV-229E, HCoV-NL63, HCoV-OC43, HCoV-HKU1, HAdV, HRSV, RV, EV, HBoV, HMPV, HPeV | *1 HRSV* | moderate |
| Freeman  et al (2021) ^25^ | United States of America | Multicenter, retrospective analysis | COVID-19 suspected patients | 09/03/2020 to 02/10/2020 | 288 | 13 (4.5) | multiplex RT-PCR | FLUA, FLUB, HPIV-1, HPIV-2, HPIV-3, HPIV-4, HCoV-229E, HCoV-NL63, HCoV-OC43, HCoV-HKU1, HAdV, HRSV, RV or EV, HMPV | *6 HAdV*  *1 HRSV*  *6 RV* | moderate |
| Hazra  et al (2020) ^26^ | United States of America | Singlecenter, retrospective cohort study | patients with influenza-like illness symptoms | 12/03/2020 to 15/04/2020 | 459 | 15 (3.3) | RT-PCR from nasopharyngeal swab (BioFire^®^ FilmArray^®^ RP 2.1 Panel) | FLUA, FLUB, HPIV-1, HPIV-2, HPIV-3, HPIV-4, HCoV-229E, HCoV-NL63, HCoV-OC43, HCoV-HKU1, HAdV, HRSV, RV or EV, HMPV, MERS-CoV | *3 FLUA*  *1 HPIV-2*  *1 HCoV-NL62*  *2 HAdV*  *8 RV or EV*  *2 HMPV* | moderate |
| Hirotsu  et al (2020) ^27^ | Japan | Multicenter, prospective cohort study | COVID-19 suspected patients | 10/03/2020 to 07/05/2020 | 8 | 0 (0.0) | multiplex PCR from nasopharyngeal swab (FilmArray Respiratory Panel) | FLUA, FLUB, HPIV-1, HPIV-2, HPIV-3, HPIV-4, HCoV-229E, HCoV-NL63, HCoV-OC43, HCoV-HKU1, HAdV, HRSV, RV or EV, HMPV, MERS-CoV | *NA* | moderate |
| Hughes  et al (2020) ^28^ | United Kingdom | Multicenter, retrospective cohort study | COVID-19 confirmed admitted adult patients | 20/02/2020 to 20/04/2020 | 250 | 0 (0.0) | RT-PCR panel from naso- or oropharyngeal swab | FLUA, FLUB, HRSV | *NA* | moderate |
| Jongbloed  et al (2021) ^29^ | Netherlands | Singlecenter, retrospective cohort study | admitted adult patients | 29/02/2020 to 01/04/2020 | 303 | 0 (0.0) | multiplex  RT-PCR from naso- and oropharyngeal swab, bronchoalveolar lavage (BAL) liquids or sputum collections | FLUA, FLUB, HPIV-1, HPIV-2, HRSV, HMPV | *NA* | moderate |
| Kim D  et al (2020) ^30^ | United States of America | Multicenter, retrospective cohort study | symptomatic  patients | 03/03/2020 to 25/03/2020 | 116 | 24 (20.7) | RT-PCR from  nasopharyngeal swabs | FLUA, FLUB, HPIV-1, HPIV-2, HPIV-3, HPIV-4, HCoV, HAdV, HRSV, RV or EV, HMPV | *1 FLUA*  *1 HPIV-1*  *1 HPIV-3*  *1 HPIV-4*  *5 HCoV (species not specified)*  *6 HRSV*  *8 RV or EV*  *2 HMPV* | high |
| Kim KW  et al (2021) ^31^ | Australia | Singlecenter, retrospective cohort study | COVID-19 confirmed  patients | 01/03/2020 to 31/05/2020 | 92 | 8 (8.7) | RT-PCR from  nasopharyngeal swab (Twist Respiratory Virus Panel, Virome Capture  Sequencing) | FLUA, FLUB, HPIV-1, HPIV-2, HPIV-3, HPIV-4, HCoV-229E, HCoV-NL63, HCoV-OC43, HCoV-HKU1, HAdV, HRSV, RV, EV, HBoV, HMPV, MERS-CoV, HPeV | *2 FLUA*  *1 HAdV*  *5 RV* | moderate |
| Kim Z  et al (2021) ^32^ | Korea | Singlecenter, retrospective cohort study | COVID-19 suspected patients | 28/02/2020 to 02/05/2020 | 123 | 6 (4.9) | RT-PCR from nasopharyngeal swab (Allplex 2019‐nCoV Assay) | FLUA, FLUB, HPIV-1, HPIV-2, HPIV-3, HPIV-4, HCoV-229E, HCoV-NL63, HCoV-OC43, HCoV-HKU1, HAdV, HRSV, RV, EV, HBoV, HMPV | *1 FLUA*  *1 HCoV-229E*  *2 HAdV*  *2 RV* | high |
| Kıymet  et al (2021) ^33^ | Turkey | Singlecenter, retrospective cross-sectional study | COVID-19 suspected  children | 11/03/2020 to 11/03/2021 | 122 | 10 (8.2) | RT-PCR from nasopharyngeal swab (Bosphore Respiratory Pathogens Panel Kit V4) | FLUA, FLUB, HPIV-1, HPIV-2, HPIV-3, HPIV-4, HCoV-229E, HCoV-NL63, HCoV-OC43, HCoV-HKU1, HAdV, HRSV, RV, EV, HBoV, HMPV, HPeV | *1 HAdV*  *1 HRSV*  *6 RV*  *1 EV*  *1 HMPV* | moderate |
| Leuzinger  et al (2020) ^34^ | Switzerland | Singlecenter, prospective cohort study | patients presenting with influenza-like illness to the outpatient department or emergency department | 01/01/2020 to 29/03/2020 | 148 | 17 (11.5) | RT-PCR from naso- or oropharyngeal swab (BioFire^®^ FilmArray^®^ RP 2.1 Panel) | FLU, HPIV, HCoV, HAdV, HRSV, RV, EV, HMPV, MERS-CoV | *2 FLU (genus not specified)*  *4 HPIV (species not specified)*  *5 HCoV (species not specified)*  *1 HAdV*  *1 HRSV*  *7 RV* | moderate |
| Li  et al (2021) ^35^ | China | Singlecenter, retrospective case series | COVID-19 confirmed admitted children | 21/01/2020 to 16/02/2020 | 81 | 5 (6.2) | direct  immunofluorescence from sputum culture | FLUA, FLUB, HPIV-1, HPIV-2, HPIV-3, HPIV-4, HAdV, HRSV | *1 FLUA*  *2 FLUB*  *1 HAdV*  *1 HRSV* | high |
| Lin  et al (2020) ^36^ | China | Singlecenter, retrospective cohort study | COVID-19  suspected  patients | 20/01/2020 to 10/02/2020 | 92 | 6 (6.5) | RT-PCR from nasal or throat swab or sputum (multiplex rapid detection kit 2.0, Uni-MEDICA Tech) | FLUA, FLUB, HPIV-1, HPIV-2, HPIV-3, HPIV-4, HCoV-229E, HCoV-NL63, HCoV-OC43, HCoV-HKU1, HAdV, HRSV, RV, HBoV, HMPV | *1 HPIV-2*  *2 HCoV-HKU1*  *3 HRSV*  *2 RV*  *2 HMPV* | high |
| Ma  et al (2020) ^37^ | China | Singlecenter, retrospective comparative study | COVID-19 confirmed  adult patients | 28/01/2020 to 29/02/2020 | 95 | 46 (48.4) | indirect immunofluorescence assay of IgM antibodies | FLUA, FLUB | *44 FLUA*  *2 FLUB* | high |
| Marshall  et al (2021) ^38^ | Canada | Singlecenter, retrospective analysis | COVID-19 suspected patients | 28/03/2020 to 28/05/2020 | 1.141 | 39 (3.4) | PCR from respiratory specimens (NxTAG Respiratory Pathogen Panel) | FLUA, FLUB, HPIV-1, HPIV-2, HPIV-3, HPIV-4, HCoV-229E, HCoV-NL63, HCoV-OC43, HCoV-HKU1, HAdV, HRSV, RV or EV, HBoV, HMPV | *1 FLUA*  *1 HPIV-2*  *2 HCoV-NL63*  *8 HAdV*  *25 RV or EV*  *1 HMPV* | moderate |
| Masse  et al (2021) ^39^ | France | Multicenter, prospective cohort study | patients aged  eight years or older consulting for an ARI episode | 06/06/2020 to 19/01/2021 | 53 | 3 (5.7) | RT-PCR from oropharyngeal swab (Bosphore Respiratory Pathogens Panel Kit) | FLUA, FLUB, HPIV-1, HPIV-2, HPIV-3, HPIV-4, HCoV-229E, HCoV-NL63, HCoV-OC43, HCoV-HKU1, HAdV, HRSV, RV, EV, HBoV, HMPV, HPeV | *1 HPIV-1*  *1 HCoV-HKU1*  *1 RV* | moderate |
| Massey  et al (2020) ^40^ | United States of America | Multicenter, retrospective cohort study | symptomatic  patients | 25/03/2020 to 23/04/2020 | 1.690 | 36 (2.1) | RT-PCR from nasopharyngeal swab (Respira-ID™) | FLUA, FLUB, HPIV-1, HPIV-2, HPIV-3, HPIV-4, HCoV-NL63, HCoV-OC43, HCoV-HKU1, HAdV, HRSV, RV, EV, HBoV, HMPV, HPeV | *9 HAdV*  *27 HMPV* | moderate |
| Matos  et al (2020) ^41^ | Brazil | Multicenter, prospective cohort study | COVID-19 suspected  patients | 01/02/2020 to 31/03/2020 | 29 | 2 (6.9) | RT-PCR from nasopharyngeal swab (XGEN multiplex  platform) | FLUA, FLUB, HPIV-1, HPIV-2, HPIV-3, HPIV-4, HCoV-229E, HCoV-NL63, HCoV-OC43, HCoV-HKU1, HAdV, HRSV, RV, EV, HBoV, HMPV, HPeV | *2 RV* | moderate |
| Mehta  et al (2021) ^42^ | India | Singlecenter, retrospective cohort study | COVID-19 confirmed  patients | 01/04/2020 to 31/05/2020 | 50 | 8 (16.0) | next-generation sequencing from nasopharyngeal swab (Illumina Respiratory Virus Oligo Panel) | FLUA, FLUB, HPIV-1, HPIV-2, HPIV-3, HPIV-4, HCoV-229E, HCoV-NL63, HCoV-OC43, HCoV-HKU1, HAdV, HRSV, RV, EV, HBoV, HMPV, HPeV | *6 FLUA*  *2 HCoV-229E* | moderate |
| Nowak  et al (2020) ^43^ | United States of America | Multicenter, retrospective cohort study | COVID-19 suspected  patients | 16/03/2020 to 20/04/2020 | 1.204 | 36 (3.0) | (FilmArray®  Respiratory Panel, Cepheid Xpert® Xpress Flu/RSV) | FLUA, FLUB, HPIV-1, HPIV-2, HPIV-3, HPIV-4, HCoV-229E, HCoV-NL63, HCoV-OC43, HCoV-HKU1, HAdV, HRSV, RV, EV, HMPV, MERS-CoV | *1 FLUA*  *4 HCoV-229E*  *7 HCoV-NL63*  *1 HCoV-OC43*  *5 HCoV-HKU1*  *2 HAdV*  *4 HRSV*  *8 RV or EV*  *4 HMPV* | high |
| Peci  et al (2021) ^44^ | Canada | Multicenter, retrospective cross-sectional study | COVID-19 suspected  patients | 11/01/2020 to 01/03/2020 | 325 | 8 (2.5) | multiplex RT-PCR from respiratory specimens | FLUA, FLUB, HPIV-1, HPIV-2, HPIV-3, HPIV-4, HCoV, HAdV, HRSV, RV, EV, HMPV | *2 HCoV (species not specified)*  *2 HRSV*  *2 RV*  *2 HMPV* | moderate |
| Pigny  et al (2021) ^45^ | Switzerland | Singlecenter, retrospective cohort study | COVID-19 confirmed positive  patients, children <16 years | 01/03/2020 to 30/04/2020 | 51 | 4 (7.8) | in-house RT-PCR panel or multiplex RT-PCR (FTD Respiratory Pathogens 21) | FLUA, FLUB, HPIV-1, HPIV-2, HPIV-3, HPIV-4, HCoV-229E, HCoV-NL63, HCoV-OC43, HCoV-HKU1, HAdV, HRSV, HBoV, HMPV | *2 HCoV2-NL63*  *1 HAdV*  *1 HMPV* | high |
| Pongpirul  et al (2020) ^46^ | Thailand | Singlecenter, retrospective case series | COVID-19 confirmed admitted patients | 08/01/2020 to 31/01/2020 | 11 | 2 (18.2) | RT-PCR Respiratory Panel (BioFire^®^ FilmArray^®^ Pneumonia Panel) | FLUA, FLUB, HPIV-1, HPIV-2, HPIV-3, HPIV-4, HCoV-229E, HCoV-NL63, HCoV-OC43, HCoV-HKU1, HAdV, HRSV, RV, EV, HBoV, HMPV, HPeV | *1 FLUA*  *1 HAdV* | high |
| Potdar  et al (2020) ^47^ | India | Multicenter, retrospective cohort study | overseas travellers reported to airports | 22/01/2020 to 29/02/2020 | 4 | 0 (0.0) | RT-PCR from throat or nasal swab | FLUA, FLUB, HPIV-1, HPIV-2, HPIV-3, HPIV-4, HCoV-229E, HCoV-NL63, HCoV-OC43, HCoV-HKU1, HAdV, HRSV, RV, HMPV | *NA* | moderate |
| Richardson et al (2020) ^48^ | United States of America | Multicenter, retrospective case series | COVID-19 confirmed patients | 01/03/2020 to 04/04/2020 | 1.996 | 39 (2.0) | RT-PCR from  nasopharyngeal swabs | FLUA, FLUB, HPIV-1, HPIV-2, HPIV-3, HPIV-4, HCoV, HAdV, HRSV, RV or EV, HMPV | *1 FLUA*  *3 HPIV-3*  *7 HCoV (species not specified)*  *4 HRSV*  *22 RV or EV*  *2 HMPV* | moderate |
| Rodriguez  et al (2021) ^49^ | France | Singlecenter, prospective cohort study | COVID-19 confirmed patients | 09/03/2020 to 30/03/2020 | 104 | 7 (6.7) | Metagenomics  analysis | FLUA, FLUB, HPIV-1, HPIV-2, HPIV-3, HPIV-4, HCoV-229E, HCoV-NL63, HCoV-OC43, HCoV-HKU1, HAdV, HRSV, RV, EV, HBoV, HMPV, MERS-CoV, HPeV | *2 FLUB*  *2 HAdV*  *1 HRSV*  *2 RV* | high |
| Roh  et al (2021) ^50^ | Korea | Singlecenter, retrospective cohort study | COVID-19 confirmed  admitted  adult patients | 09/02/2020 to 23/02/2020 | 342 | 27 (7.9) | multiplex  RT-PCR from naso- or, oropharyngeal swab or sputum specimens  (Allplex TM Respiratory Panel 1 and 2) | FLUA, FLUB, HPIV-1, HPIV-2, HPIV-3, HPIV-4, HCoV-229E, HCoV-NL63, HCoV-OC43, HCoV-HKU1, HAdV, HRSV, RV, EV, HBoV, HMPV | *3 FLUA*  *3 HCoV-229E*  *1 HCoV-NL63*  *1 HCoV-OC43*  *3 HAdV*  *6 HRSV*  *7 RV*  *1 HBoV*  *4 HMPV* | high |
| Schneider  et al (2021) ^51^ | United States of America | Multicenter, retrospective cohort study | symptomatic children | 25/03/2020 to 17/05/2020 | 18 | 4 (22.2) | RT-PCR from nasopharyngeal swab (FilmArray  Respiratory Panel 2) | FLUA, FLUB, HPIV-1, HPIV-2, HPIV-3, HPIV-4, HCoV-229E, HCoV-NL63, HCoV-OC43, HCoV-HKU1, HAdV, HRSV, RV or EV, HMPV, MERS-CoV | *1 HPIV-4*  *2 HCoV-NL63*  *1 HRSV*  *2 RV or EV* | high |
| Shah  et al (2020) ^52^ | United States of America | Singlecenter, retrospective cohort study | emergency department adult patients with an acute respiratory illness | 03/02/2020 to 31/03/2020 | 27 | 0 (0.0) | RT-PCR from naso- or oropharyngeal swab (Cepheid Xpert® Xpress Flu/RSV) and metagenomic next generation sequencing | FLUA, FLUB, HPIV-1, HPIV-2, HPIV-3, HPIV-4, HCoV-229E, HCoV-NL63, HCoV-OC43, HCoV-HKU1, HAdV, HRSV, RV, EV, HBoV, HMPV | *NA* | moderate |
| Si  et al (2020) ^53^ | China | Singlecenter, retrospective cohort study | COVID-19 suspected patients | 21/01/2020 to 29/02/2020 | 24 | 1 (4.2) | RT-PCR from nasopharyngeal or throat swab or sputum specimens | FLUA, FLUB, HPIV, HCoV-229E, HCoV-NL63, HCoV-OC43, HCoV-HKU1, HAdV, HRSV, RV, HBoV, HMPV | *1 HPIV (species not specified)* | moderate |
| Singh  et al (2021) ^54^ | United States of America | Singlecenter, retrospective cohort study | NA | 16/03/2020 to 01/08/2020 | 4.259 | 47 (1.1) | RT-PCR from  nasal, oropharyngeal or sputum swab | FLU, HPIV, HCoV, HAdV, HRSV, RV, EV, HMPV | *1 FLU (genus not specified)*  *2 HPIV (species not specified)*  *3 HCoV (species not specified)*  *13 HAdV*  *5 HRSV*  *21 RV*  *2 HMPV* | moderate |
| Søgaard  et al (2021) ^55^ | Switzerland | Singlecenter, retrospective descriptive study | COVID-19 confirmed admitted  patients | 25/02/2020 to 31/05/2020 | 87 | 5 (5.7) | PCR from nasopharynx, sputum, tracheal secrete, and bronchoalveolar lavage fluid (BioFire^®^ FilmArray^®^ Pneumonia Panel plus) | FLUA, FLUB, HPIV-1, HPIV-2, HPIV-3, HPIV-4, HCoV-229E, HCoV-NL63, HCoV-OC43, HCoV-HKU1, HAdV, HRSV, RV or EV, HMPV, MERS-CoV | *1 FLUA*  *1 HPIV-3*  *1 HCoV-HKU1*  *1 HRSV*  *1 RV or EV* | high |
| Tong  et al (2021) ^56^ | China | Singlecenter, retrospective cohort study | COVID-19 confirmed patients | 08/02/2020 to 15/03/2020 | 140 | 73 (52.1) | IgM immunofluorescence assay from peripheral blood samples | FLU | *73 FLU (genus not specified)* | high |
| Wang  et al (2020) ^57^ | China | Singlecenter, retrospective cohort study | COVID-19  suspected  patients | 20/01/2020 to 09/02/2020 | 104 | 6 (5.8) | respiratory electrophoresis fragment analysis with PCR from sputum samples | FLUA, FLUB, HPIV-1, HPIV-2, HPIV-3, HPIV-4, HCoV, HAdV, HRSV, RV, HBoV, HMPV | *3 FLUA*  *3 HCoV (species not specified)*  *2 RV* | moderate |
| Wei  et al (2020) ^58^ | China | Singlecenter, retrospective cohort study | COVID-19 confirmed childbearing-age women patients | 19/01/2020 to 02/03/2020 | 43 | 12 (27.9) | enzyme-linked immunosorbent assay (ELISA) for IgM from throat swabs | FLUA, FLUB, HPIV-1, HPIV-2, HPIV-3, HPIV-4, HAdV, HRSV | *12 FLUA* | moderate |
| Wu  et al (2020) ^59^ | China | Singlecenter, retrospective cohort study | COVID-19 confirmed  admitted patients | 25/12/2019 to 26/01/2020 | 173 | 1 (0.6) | RT-PCR from throat swab | FLUA, FLUB, HPIV-1, HPIV-2, HPIV-3, HPIV-4, HAdV, HRSV | *1 FLUA* | moderate |
| Xing  et al (2020) ^60^ | China | Multicenter, retrospective cohort study | COVID-19 confirmed patients | 17/01/2020 to 16/02/2020 | 68 | 25 (36.8) | Indirect immunofluorescence for IgM antibodies and RT-PCR from throat swab | FLUA, FLUB, HAdV, HRSV | *18 FLUA*  *16 FLUB*  *1 HRSV* | moderate |
| Zhu  et al (2020) ^61^ | China | Singlecenter, retrospective cohort study | COVID-19 confirmed patients | 22/01/2020 to 02/02/2020 | 257 | 31 (12.1) | RT-PCR from throat swab | FLUA, FLUB, HPIV-1, HPIV-2, HPIV-3, HPIV-4, HCoV-229E, HCoV-NL63, HCoV-OC43, HCoV-HKU1, HAdV, HRSV, RV, HBoV, HMPV | *2 FLUA*  *5 FLUB*  *10 HAdV*  *12 RV*  *1 HBoV*  *1 HMPV* | high |

Abbreviations: FLU: Human influenza virus, FLUA: Human influenza virus A, FLUB: Human influenza virus B, HPIV: Human parainfluenza virus, HPIV-1: Human parainfluenza virus 1, HPIV-2: Human parainfluenza virus 2, HPIV-3: Human parainfluenza virus 3, HPIV-4: Human parainfluenza virus 4, HCoV: Human respiratory coronavirus, HCoV-229E: Human respiratory coronavirus 229E strain, HCoV-NL63: Human respiratory coronavirus NL63 strain, HCoV-OC43: Human respiratory coronavirus OC43 strain, HCoV-HKU1: Human respiratory coronavirus HKU1 strain, HAdV: Human adenovirus, HRSV: Human respiratory syncytial virus, RV: Human rhinovirus, EV: Human enterovirus, HBoV: Human bocavirus, HMPV: Human metapneumovirus, MERS-CoV: Middle East respiratory syndrome-related coronavirus, HPeV: Human parechovirus; SARS-CoV-2: Severe acute respiratory syndrome-related coronavirus 2; COVID-19: coronavirus disease 2019; NA: not available

**Supplementary Table S4.** GRADE Evidence Profile ^62^ - Grading of Recommendations, Assessment, Development, and Evaluations

Overall Quality of Evidence for Pooled Prevalence of Respiratory Co-infections

| **Certainty Assessment** | | | | | | **Other**  **considerations** | **Total**  **Patients**  ***studies*** | **Effect**  **Pooled Prevalence**  **95% CI** | **Grade Quality of Evidence** |
| --- | --- | --- | --- | --- | --- | --- | --- | --- | --- |
| **Study**  **design** | **Risk of Bias*** | **Inconsistency^†^** | **Indirect****ness‡** | **Imprecision***^§^* | **Publication**  **Bias**** |  |  |  |  |
| Observational  Studies | Not  Serious | Serious | Not  Serious | Not  Serious | Not  Serious | all plausible residual confounding would reduce the demonstrated effect | 16.643  *59 studies* | 0.05  (0.03-0.07) | **🞅🞅⮿⮿**  **Low** |

CI: confidence interval

**Initial and Final Grade**

Step 1 – Initial low quality due to mostly observational studies. Current meta-analyses included mostly observational studies which equates to initial low quality. 🞅🞅⮿⮿

Step 2 - Downgraded 1 level for Inconsistency.

Step 3 – Upgraded 1 levels for residual confounding and biases.

**Step 1 - Evaluation of initial Grade**

Study design - Current meta-analyses included all type of studies with at least 10 participants, which were mostly observational studies (52/59), six case series and one experimental study and equates to initial low quality.

**Step 2 - Subsequent evaluation To Lower Grade**

* Risk of Bias - No serious risk of bias since 37/59 (63%) of studies had a Newcastle Ottawa Scale of 6-7 (moderate study quality) and 22/59 (37%) of 8-9 (high study quality).

**^†^** Inconsistency - Serious inconsistency (substantial heterogeneity) since I^2^ is > 0.9 (0,95).

**‡** Indirectness **-** No serious risk of indirectness through differences in populations since no major restrictions on population were applied. Differences in interventions (test or respiratory viral co-infections) since different panels were used to screen for respiratory viral co-infections and testing was not uniformly performed for all respiratory viral co-pathogens may cause a risk of indirectness, but no serious risk.

^§^ Imprecision – Imprecision was not present due to a very large sample size (N=16.643).

^**^ Publication bias – Not serious since the Egger test (p-value= 0.7820) and Begg’s correlation (p-value= 0.2092) did not show any significant publication bias.

**Step 3 - Subsequent evaluation to Increase Grade**

Quality score can be raised the based on the 3 domains of (Large Effect, Dose Response, Plausible Residual Confounding/Bias).

- Large effects - No increase in quality for this study can be made for this factor.
- Dose Response - No increase in quality for this study can be made for this factor.
- Residual confounding and biases - Upgrade since this factor works to reduce (underestimate) the demonstrated effect. The true effect may be greater than the demonstrated effect since not all patients were tested for co-infections (only suspected or confirmed COVID-19 cases, asymptomatic patients were not taken into account) and testing was not uniformly performed for all respiratory viral co-pathogens.

**Supplementary Table S5.** Summary of Findings of Secondary Outcome Sub-analyses

| **Certainty Assessment** | | | | | | | | **Summary of Findings** | | | | **Grade Quality of Evidence** |
| --- | --- | --- | --- | --- | --- | --- | --- | --- | --- | --- | --- | --- |
| **Total**  **Studies** | **Study**  **design** | **Risk of Bias** | **Incon- sistency** | **Indirect-**  **ness** | **Impreci-**  **sion** | **Publication**  **Bias** | **Other**  **considerations** | **Events**  **co-infected group** | **Events mono-infected group** | **Relative Effect (95% CI)** | **Absolute**  **Effect (95% CI)** |  |
| **Occurrence of symptom COUGH in the mono-infected vs. co-infected subgroup** | | | | | | | | | | | | |
| 5 | Obser-  vational  Studies | Not  Serious | Not  Serious  (I^2^=8%) | Not  Serious | Not  (OIS= 7.679) | Not  Serious  (Eggers  p= 0.6335,  Begg´s  p= 0.3272) | all plausible residual confounding would reduce the demonstrated effect | 82/131 (62.6%) | 136/225 (60.4%) | **OR 0.72** (0.42 to 1.21) | **81 fewer per 1.000** (from 214 fewer to 45 more) | **🞅🞅⮿⮿**  **Low** |
| **Occurrence of symptom FEVER in the mono-infected vs. co-infected subgroup** | | | | | | | | | | | | |
| 5 | Obser-  vational  Studies | Not  Serious | Not  Serious  (I^2^=28%) | Not  Serious | Serious  (OIS= 5.585) | Not  Serious  (Eggers  p= 0.8535,  Begg´s  p= 1.0000) | all plausible residual confounding would reduce the demonstrated effect | 96/131 (73.3%) | 171/225 (76.0%) | **OR 0.62** (0.35 to 1.12) | **97 fewer per 1.000** | **🞅🞅⮿⮿**  **Low** |
| **Occurrence of symptom DYSPNEA in the mono-infected vs. co-infected subgroup** | | | | | | | | | | | | |
| 5 | Obser-  vational  Studies | Not  Serious | Not  Serious  (I^2^=41%) | Not  Serious | Serious  (OIS= 329) | Not  Serious  (Eggers  p= 0.8621,  Begg´s  p= 0.6242) | all plausible residual confounding would reduce the demonstrated effect | 63/131 (48.1%) | 84/225 (37.3%) | **OR 0.97** (0.38 to 2.47) | **7 fewer per 1.000** (from 189 fewer to 222 more) | **🞅🞅⮿⮿**  **Low** |
| **ICU-Admission-Rate in the mono-infected vs. co-infected subgroup** | | | | | | | | | | | | |
| 7 | Obser-  vational  Studies | Not  Serious | Not  Serious  (I^2^=0%) | Not  Serious | Serious  (OIS= 33.0925) | Not  Serious  (Eggers  p= 0.8551,  Begg´s  p= 0.8806) | all plausible residual confounding would reduce the demonstrated effect | 20/78 (25.6%) | 228/901 (25.3%) | **OR 0.89** (0.42 to 1.87) | **21 fewer per 1.000** (from 128 fewer to 135 more) | **🞅🞅⮿⮿**  **Low** |
| **Case-Fatality-Rate in the mono-infected vs. co-infected subgroup** | | | | | | | | | | | | |
| 10 | Obser-  vational  Studies | Not  Serious | Not  Serious  (I^2^=67%) | Not  Serious | Not  Serious  (OIS= 129) | Not  Serious  (Eggers  p= 0.4894,  Begg´s  p= 0.3272) | all plausible residual confounding would reduce the demonstrated effect | 37/203 (18.2%) | 42/626 (6.7%) | **OR 1.66** (0.40 to 6.78) | **40 more per 1.000** (from 39 fewer to 261 more) | **🞅⮿⮿⮿**  **Moderate** |
| **Proportion of male gender in the mono-infected vs. co-infected subgroup** | | | | | | | | | | | | |
|  | | | | | | | | Co-infections among Males | Co-infections among Females |  | | |
| 12 | Obser-  vational  Studies | Not  Serious | Not  Serious  (I^2^=0%) | Not  Serious | Not  Serious  (OIS= 28.447) | Not  Serious  (Eggers  p= 0.3061,  Begg´s  p= 0.2726) | all plausible residual confounding would reduce the demonstrated effect | 138/1357 (10.2%) | 112/1182 (9.5%) | **OR 0.79** (0.59 to 1.06) | **18 fewer per 1.000** (from 37 fewer to 5 more) | **🞅🞅⮿⮿**  **Low** |

CI: confidence interval; OR: Odds Ratio, OIS: optimal information size, ICU: intensive care unit

**Supplementary Table S6.** PRISMA ^63,64^ Checklist

| **Section and Topic** | **Item #** | **Checklist item** | **Page where item is reported** |
| --- | --- | --- | --- |
| **TITLE** | | |  |
| Title | 1 | Identify the report as a systematic review. | 1 |
| **ABSTRACT** | | |  |
| Abstract | 2 | See the PRISMA 2020 for Abstracts checklist. | 2 |
| **INTRODUCTION** | | |  |
| Rationale | 3 | Describe the rationale for the review in the context of existing knowledge. | 3 |
| Objectives | 4 | Provide an explicit statement of the objective(s) or question(s) the review addresses. | 3 |
| **METHODS** | | |  |
| Eligibility criteria | 5 | Specify the inclusion and exclusion criteria for the review and how studies were grouped for the syntheses. | 4 |
| Information sources | 6 | Specify all databases, registers, websites, organisations, reference lists and other sources searched or consulted to identify studies. Specify the date when each source was last searched or consulted. | 4 |
| Search strategy | 7 | Present the full search strategies for all databases, registers and websites, including any filters and limits used. | 4 |
| Selection process | 8 | Specify the methods used to decide whether a study met the inclusion criteria of the review, including how many reviewers screened each record and each report retrieved, whether they worked independently, and if applicable, details of automation tools used in the process. | 4 |
| Data collection process | 9 | Specify the methods used to collect data from reports, including how many reviewers collected data from each report, whether they worked independently, any processes for obtaining or confirming data from study investigators, and if applicable, details of automation tools used in the process. | 4 |
| Data items | 10a | List and define all outcomes for which data were sought. Specify whether all results that were compatible with each outcome domain in each study were sought (e.g. for all measures, time points, analyses), and if not, the methods used to decide which results to collect. | 4,5 |
|  | 10b | List and define all other variables for which data were sought (e.g. participant and intervention characteristics, funding sources). Describe any assumptions made about any missing or unclear information. | 4,5 |
| Study risk of bias assessment | 11 | Specify the methods used to assess risk of bias in the included studies, including details of the tool(s) used, how many reviewers assessed each study and whether they worked independently, and if applicable, details of automation tools used in the process. | 4 |
| Effect measures | 12 | Specify for each outcome the effect measure(s) (e.g. risk ratio, mean difference) used in the synthesis or presentation of results. | 4,5 |
| Synthesis methods | 13a | Describe the processes used to decide which studies were eligible for each synthesis (e.g. tabulating the study intervention characteristics and comparing against the planned groups for each synthesis (item #5)). | 4 |
|  | 13b | Describe any methods required to prepare the data for presentation or synthesis, such as handling of missing summary statistics, or data conversions. | 4,5 |
|  | 13c | Describe any methods used to tabulate or visually display results of individual studies and syntheses. | 4,5 |
|  | 13d | Describe any methods used to synthesize results and provide a rationale for the choice(s). If meta-analysis was performed, describe the model(s), method(s) to identify the presence and extent of statistical heterogeneity, and software package(s) used. | 5 |
|  | 13e | Describe any methods used to explore possible causes of heterogeneity among study results (e.g. subgroup analysis, meta-regression). | 5 |
|  | 13f | Describe any sensitivity analyses conducted to assess robustness of the synthesized results. | 5 |
| Reporting bias assessment | 14 | Describe any methods used to assess risk of bias due to missing results in a synthesis (arising from reporting biases). | 5 |
| Certainty assessment | 15 | Describe any methods used to assess certainty (or confidence) in the body of evidence for an outcome. | 5 |
| **RESULTS** | | |  |
| Study selection | 16a | Describe the results of the search and selection process, from the number of records identified in the search to the number of studies included in the review, ideally using a flow diagram. | 5 |
|  | 16b | Cite studies that might appear to meet the inclusion criteria, but which were excluded, and explain why they were excluded. | 5 |
| Study characteristics | 17 | Cite each included study and present its characteristics. | 5 |
| Risk of bias in studies | 18 | Present assessments of risk of bias for each included study. | 6 |
| Results of individual studies | 19 | For all outcomes, present, for each study: (a) summary statistics for each group (where appropriate) and (b) an effect estimate and its precision (e.g. confidence/credible interval), ideally using structured tables or plots. | 6,7 |
| Results of syntheses | 20a | For each synthesis, briefly summarise the characteristics and risk of bias among contributing studies. | 6,7 |
|  | 20b | Present results of all statistical syntheses conducted. If meta-analysis was done, present for each the summary estimate and its precision (e.g. confidence/credible interval) and measures of statistical heterogeneity. If comparing groups, describe the direction of the effect. | 6,7 |
|  | 20c | Present results of all investigations of possible causes of heterogeneity among study results. | 8 |
|  | 20d | Present results of all sensitivity analyses conducted to assess the robustness of the synthesized results. | 6-8 |
| Reporting biases | 21 | Present assessments of risk of bias due to missing results (arising from reporting biases) for each synthesis assessed. | 5 |
| Certainty of evidence | 22 | Present assessments of certainty (or confidence) in the body of evidence for each outcome assessed. | 7 |
| **DISCUSSION** | | |  |
| Discussion | 23a | Provide a general interpretation of the results in the context of other evidence. | 7,8 |
|  | 23b | Discuss any limitations of the evidence included in the review. | 8 |
|  | 23c | Discuss any limitations of the review processes used. | 8 |
|  | 23d | Discuss implications of the results for practice, policy, and future research. | 8 |
| **OTHER INFORMATION** | | |  |
| Registration and protocol | 24a | Provide registration information for the review, including register name and registration number, or state that the review was not registered. | 1,4 |
|  | 24b | Indicate where the review protocol can be accessed, or state that a protocol was not prepared. | 1,4 |
|  | 24c | Describe and explain any amendments to information provided at registration or in the protocol. | 1,4 |
| Support | 25 | Describe sources of financial or non-financial support for the review, and the role of the funders or sponsors in the review. | 9 |
| Competing interests | 26 | Declare any competing interests of review authors. | 9 |
| Availability of data, code and other materials | 27 | Report which of the following are publicly available and where they can be found: template data collection forms; data extracted from included studies; data used for all analyses; analytic code; any other materials used in the review. | 9 |

*From:*  Page MJ, McKenzie JE, Bossuyt PM, Boutron I, Hoffmann TC, Mulrow CD, et al. The PRISMA 2020 statement: an updated guideline for reporting systematic reviews. BMJ 2021;372:n71. doi: 10.1136/bmj.n71

For more information, visit: http://www.prisma-statement.org/

**Supplementary Figure S1.** Summary of Newcastle-Ottawa Scale Quality Assessment ^64^

**Supplementary Figure S2.** Pooled prevalence of co-infection rates among the female (A) and male (B) COVID-19 patients subgroups

**Fig. S2A Females Fig. S2B Males**







**Supplementary Figure S3.** Pooled prevalence of co-infection rates among studies including pediatric (A) or adult (B) patients only and those with no age restriction (C)

**Fig. S3A Children Fig. S3C No Age Restriction**
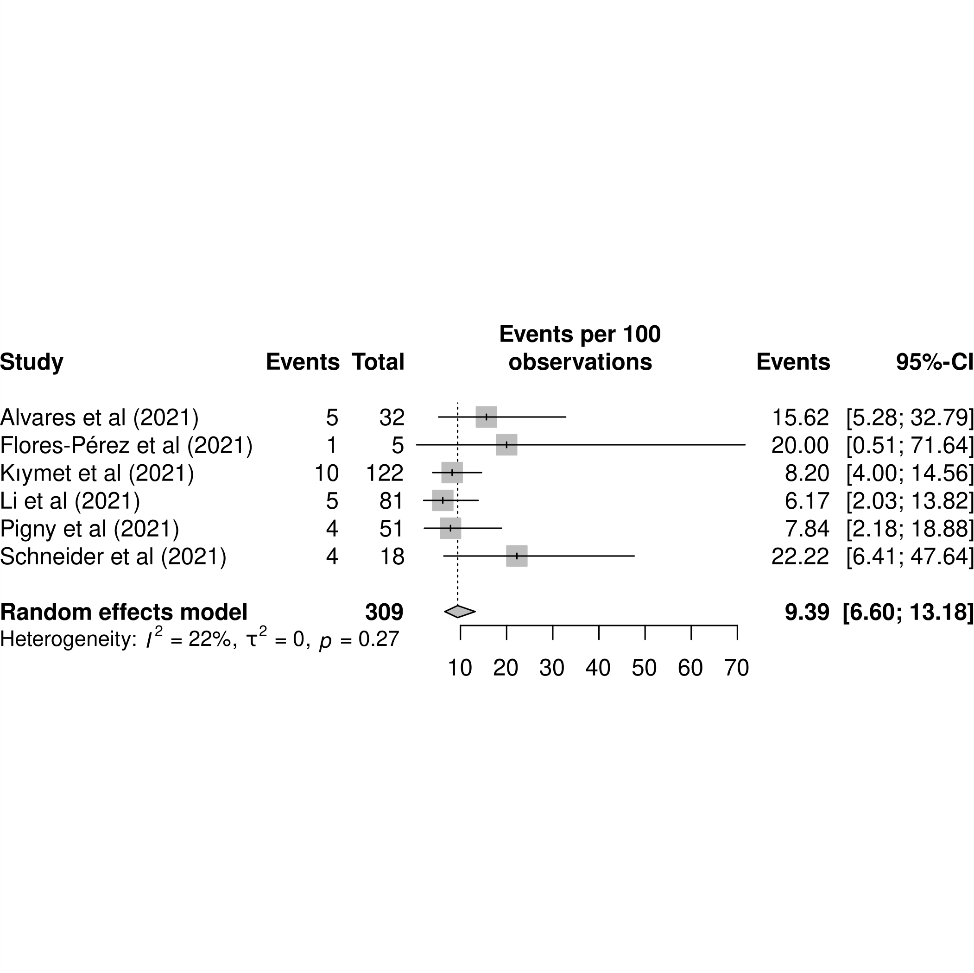


**Fig. S3B Adults**


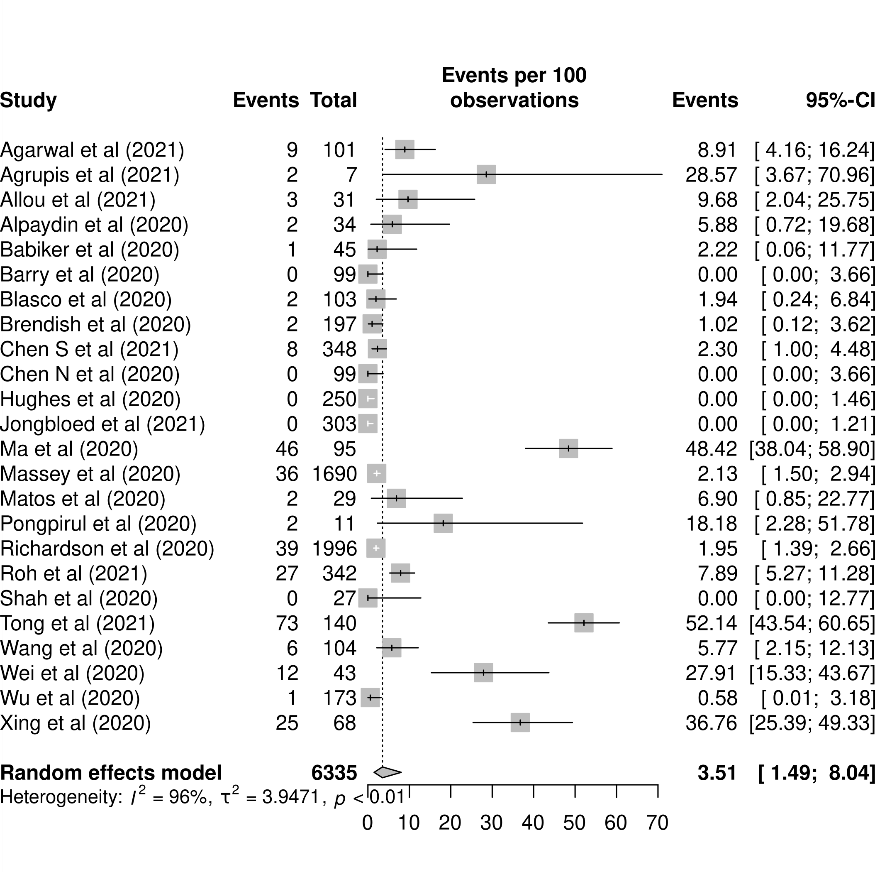

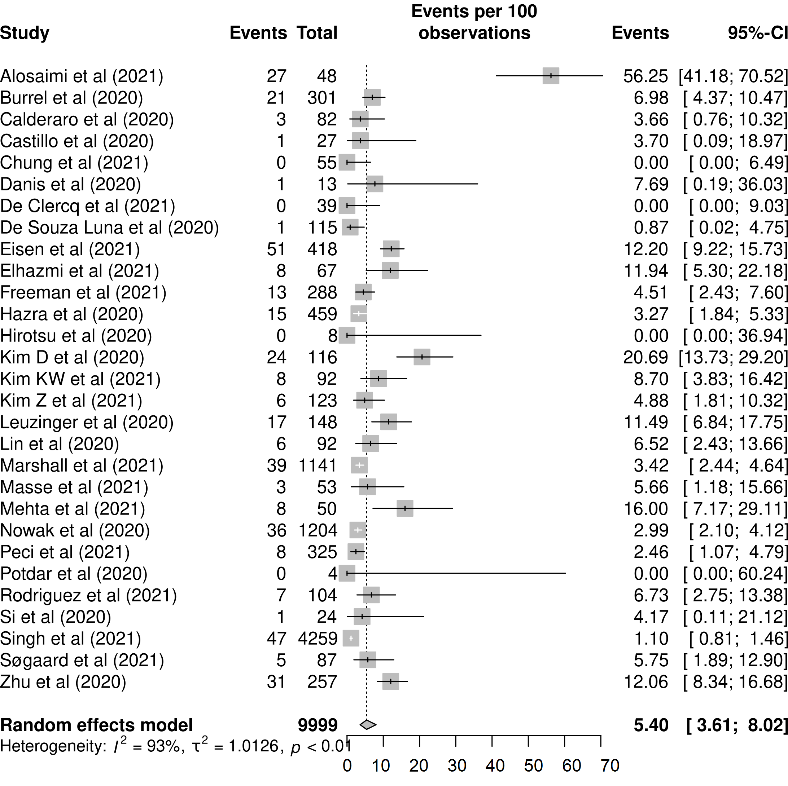


**Supplementary Figure S4.** Pooled prevalence of co-infection rates in studies that screened > 100 SARS-CoV-2 positive patients (A) and studies that screened ≥ 100 SARS-CoV-2 positive patients (B)

**Fig. S4A Small SARS-CoV-2 positive Cohort (< 100 patients) Fig. S4B Large SARS-CoV-2 positive Cohort (≥ 100 patients)**


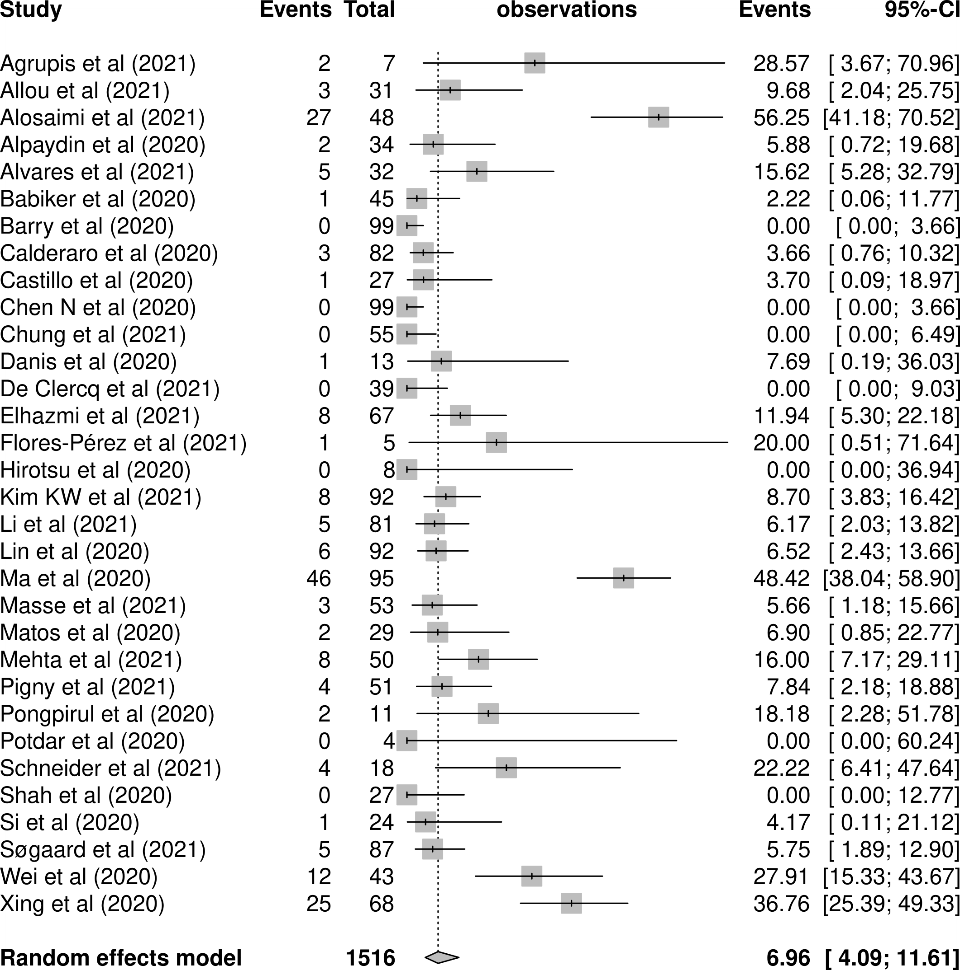

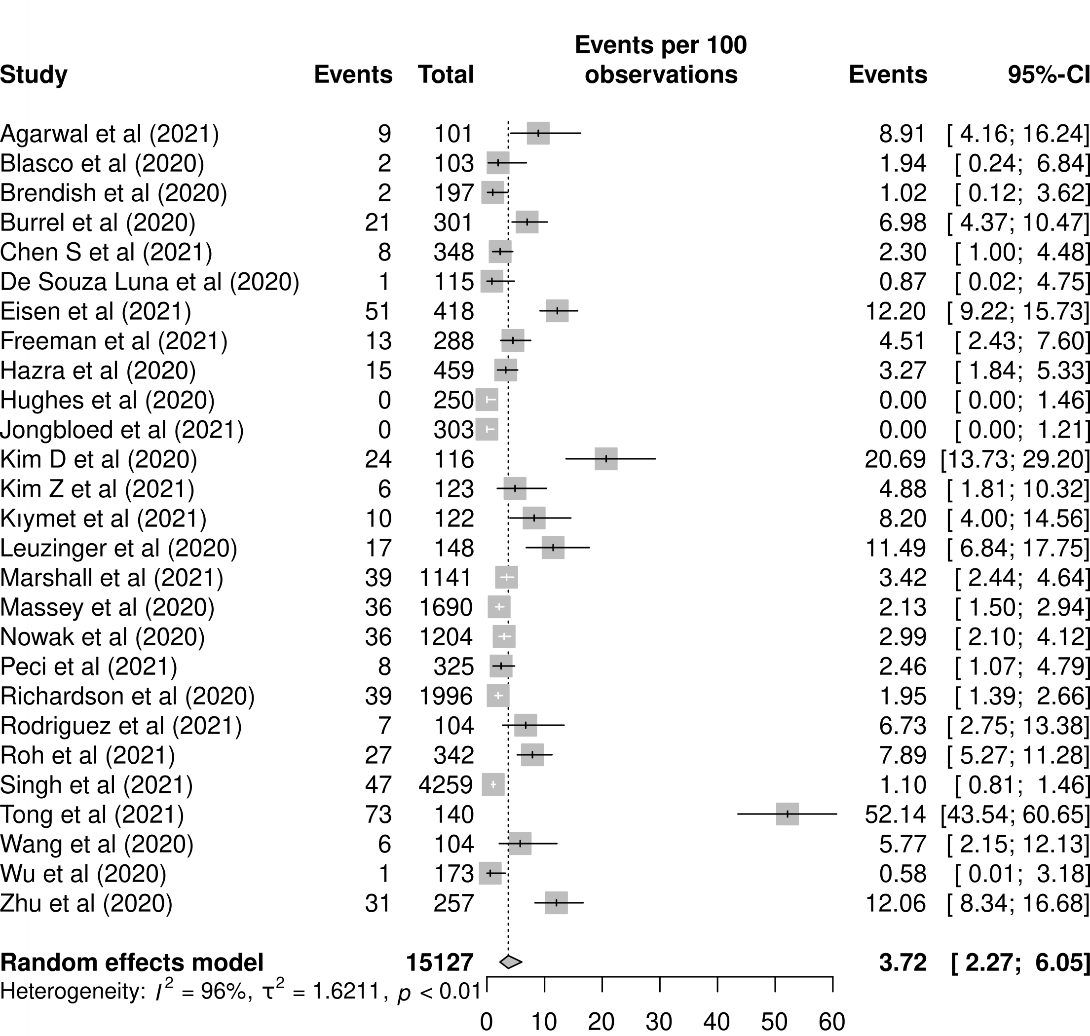


**Supplementary Figure S5.** Pooled prevalence of co-infection rates among the different continents/geographic regions Asia (A), North America (B), Europe (C) and South America (D). Studies were classified according to the United Nations “Standard Country or Area Codes for Statistical Use”. Africa and Oceania are not demonstrated, since only one study was identified for each of them.

**Fig. S5A Asia Fig. S5B North America**


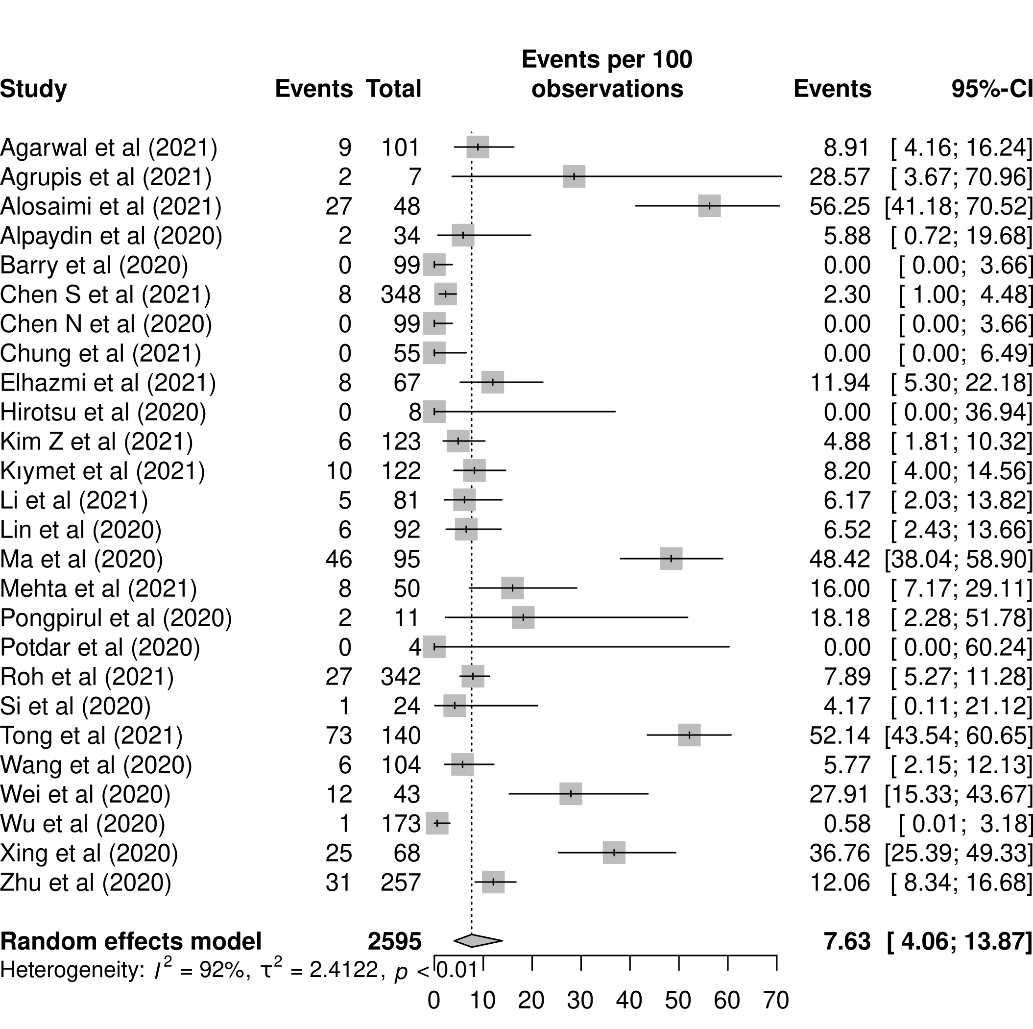




**Fig. S5C Europe Fig. S5D South America**


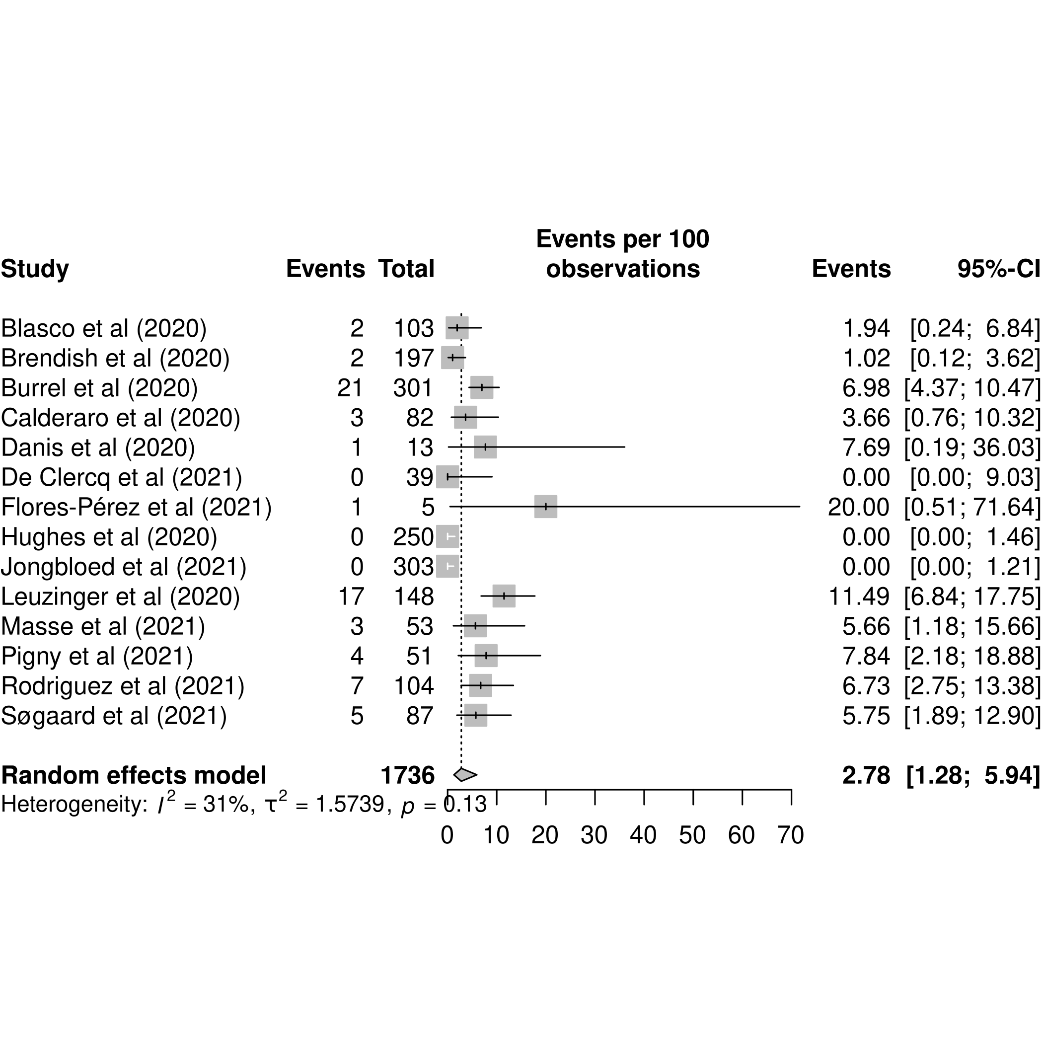




**Supplementary Figure-S6.** Pooled prevalence of co-infection rates for Spring (A), Autumn (B) or Winter (C) season. Season periods were classified according to meteorological dates. Summer is not showed, since studies that screened COVID-19 patients for co-infections during the summer months overlap with the Spring or Autumn season. Studies from tropical geographic regions (N=2) have been left out because they only experience a dry and wet season.

**Fig. S6A Spring Fig. S6C Winter**


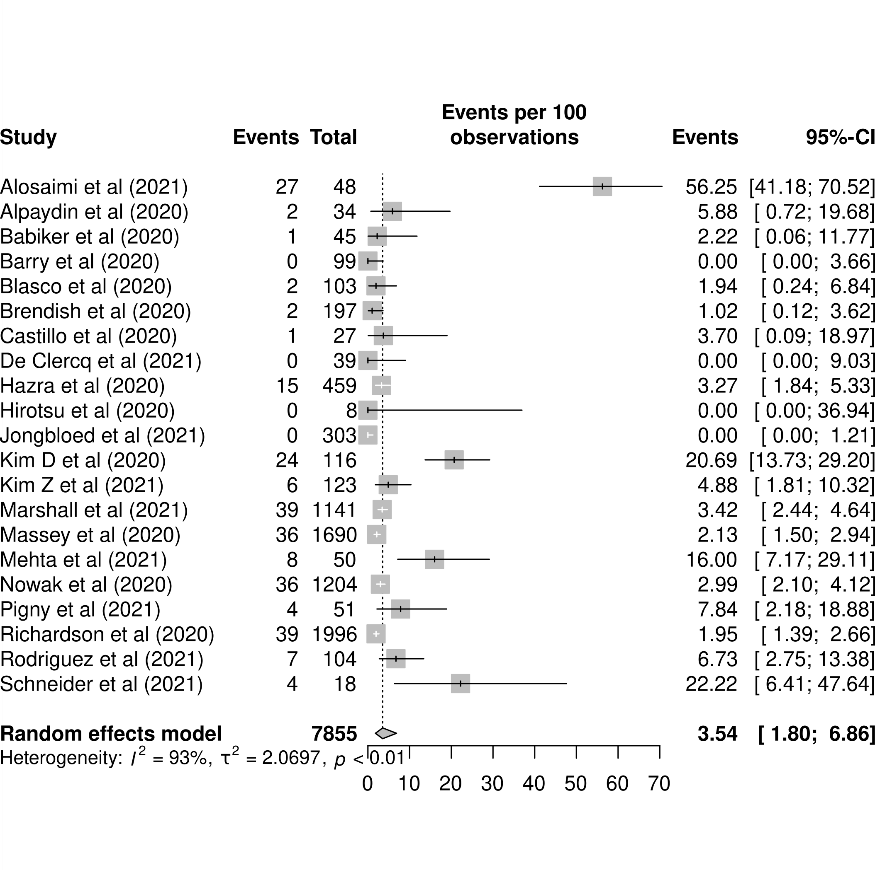

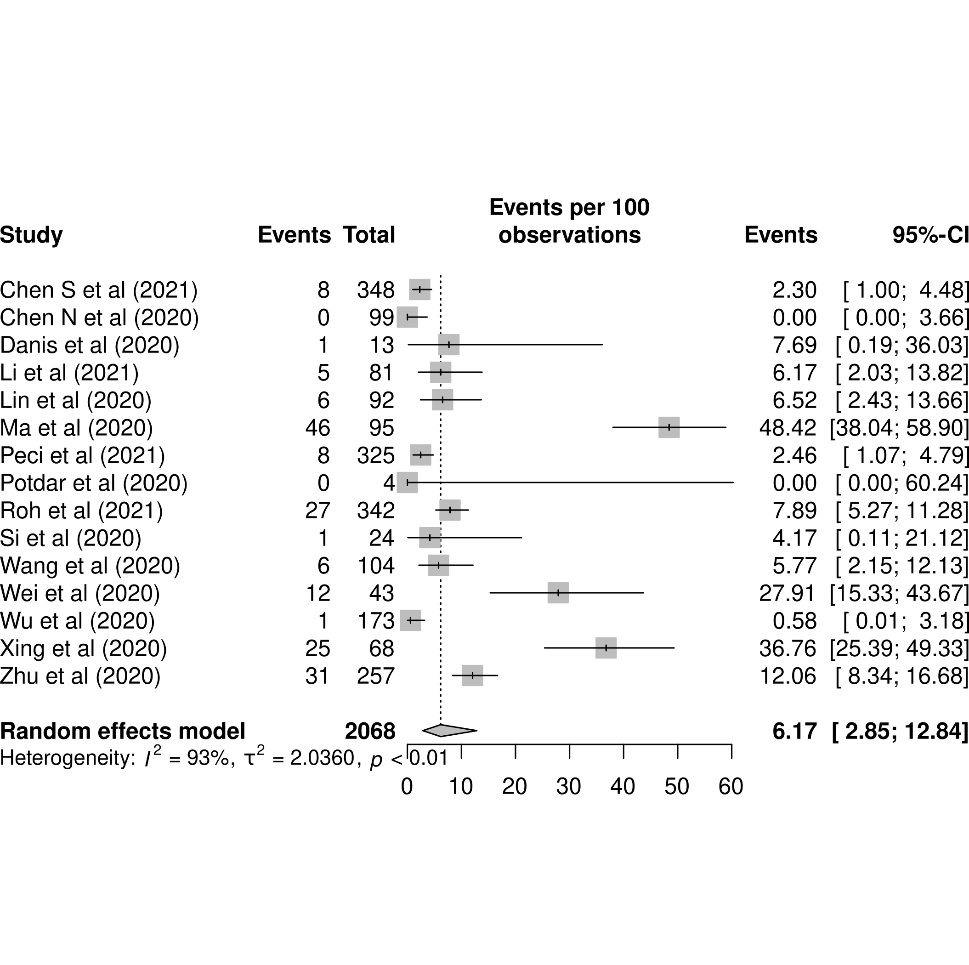


**Fig. S6B Autumn**


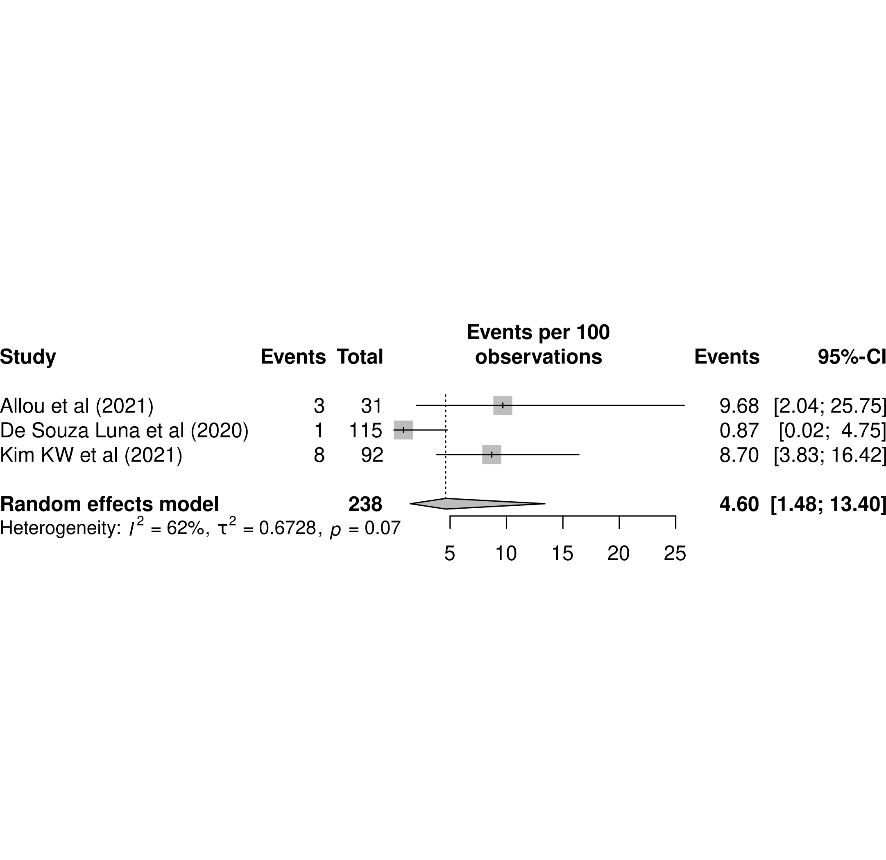


**Supplementary Figure S7.** Odds ratios of co-infection in male vs. female COVID-19 patients.
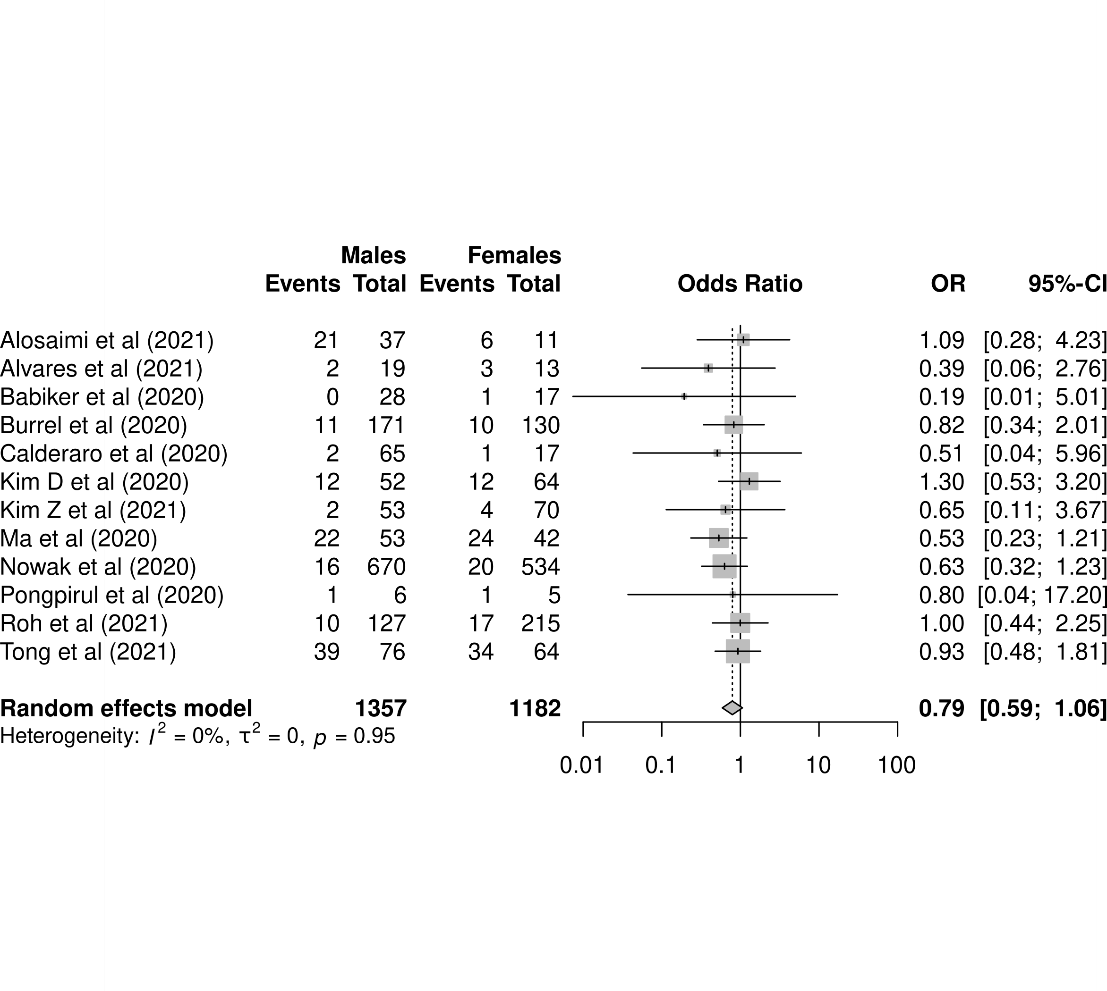


**Supplementary Figure S8.** Odds ratio of the ICU-Admission-Rate in the co-infected vs. mono-infected patient groups.





**Supplementary Figure S9.** Odds ratio of the Case-Fatality-Rate in the co-infected vs. mono-infected patient groups.


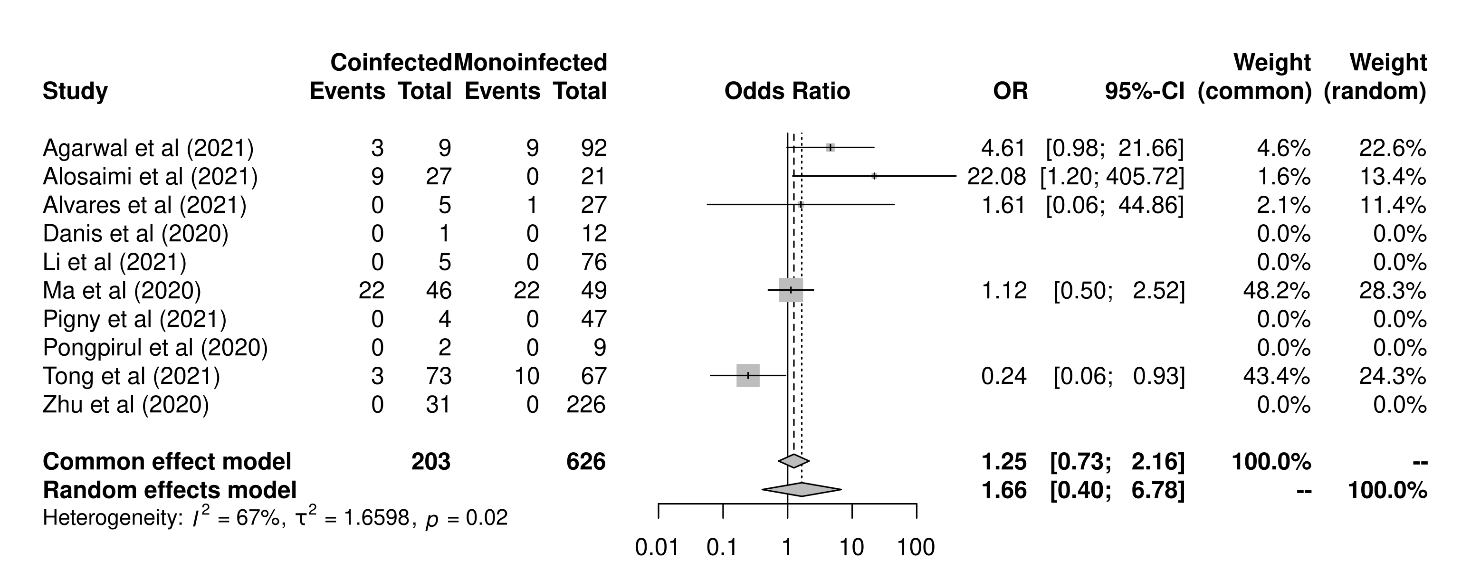


**Supplementary Figure S10.** Odds ratio of the presence of cough (A), fever (B) and dyspnea (C) in the co-infected vs. mono-infected patient group.

**Fig. S10A Cough**





**Fig. S10B Fever**


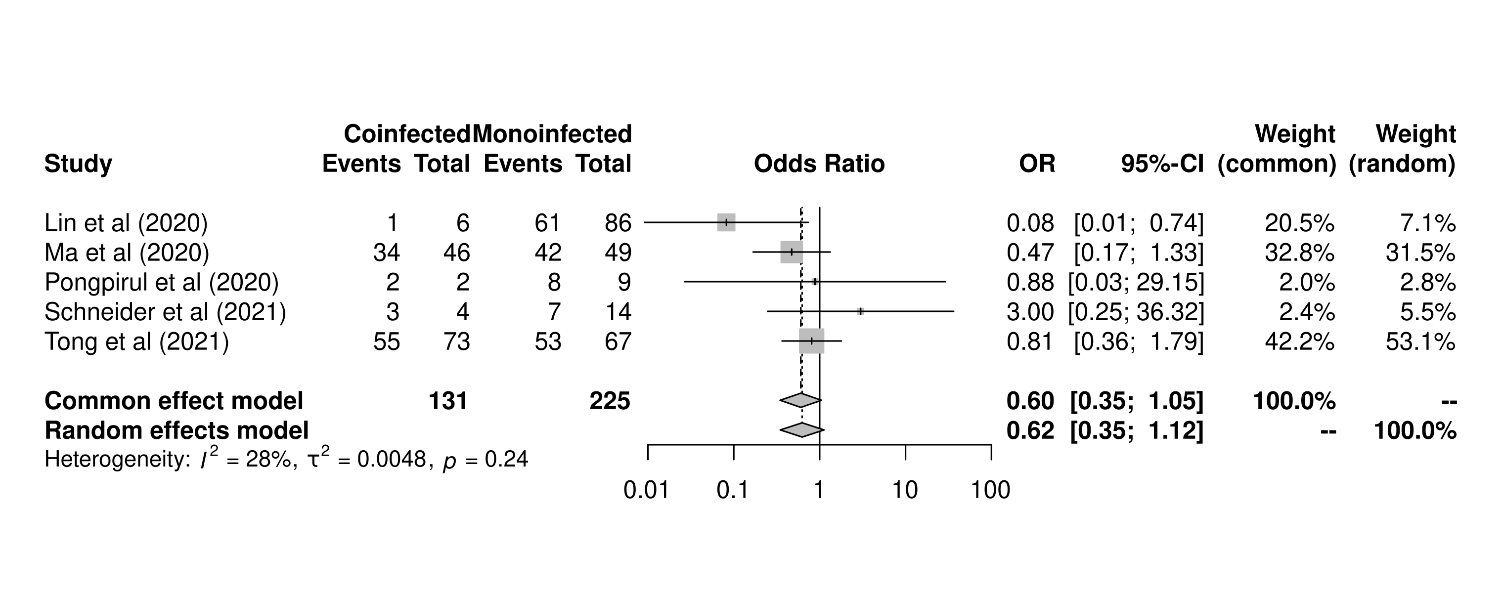


**Fig. S10C Dyspnea**





**Supplementary Figure S11.** Funnel plot on the reported co-infection rates among COVID-19 patients (p for Egger’s test=0.7820 and Begg’s test=0.2092).





References

1. WHO World Health Organisation. WHO COVID-19 Case definition: Updated in Public health surveillance for COVID-19. Accessed March 10, 2022. https://www.who.int/publications/i/item/WHO-2019-nCoV-Surveillance_Case_Definition-2020.2

2. Garner JS, Jarvis WR, Emori TG, Horan TC, Hughes JM. CDC definitions for nosocomial infections, 1988. *Am J Infect Control*. 1988;16(3):128-140. doi:10.1016/0196-6553(88)90053-3

3. Agarwal A, Agarwal M, Sharma A, Jakhar R. Impact of influenza A co-infection with COVID-19. *Int J Tuberc Lung Dis*. 2021;25(5):413-415. doi:10.5588/ijtld.21.0086

4. Agrupis K an, Villanueva AMG, Sayo AR, et al. If Not COVID-19 What Is It? Analysis of COVID-19 versus Common Respiratory Viruses among Symptomatic Health Care Workers in a Tertiary Infectious Disease Referral Hospital in Manila, Philippines. *Trop Med Infect Dis*. 2021;6(1). doi:10.3390/tropicalmed6010039

5. Allou N, Larsen K, Dubernet A, et al. Co-infection in patients with hypoxemic pneumonia due to COVID-19 in Reunion Island. *Medicine (Baltimore)*. 2021;100(4):e24524. doi:10.1097/MD.0000000000024524

6. Alosaimi B, Naeem A, Hamed ME, et al. Influenza co-infection associated with severity and mortality in COVID-19 patients. *Virol J*. 2021;18(1):127. doi:10.1186/s12985-021-01594-0

7. Alpaydin AO, Gezer NS, Simsek GO, et al. Clinical and radiological diagnosis of non-SARS-CoV-2 viruses in the era of COVID-19 pandemic. *J Med Virol*. 2021;93(2):1119-1125. doi:10.1002/jmv.26410

8. Alvares PA. SARS-CoV-2 and Respiratory Syncytial Virus Coinfection in Hospitalized Pediatric Patients. *Pediatr Infect Dis J*. 2021;40(4):e164-e166. doi:10.1097/INF.0000000000003057

9. Babiker A, Bradley HL, Stittleburg VD, et al. Metagenomic Sequencing To Detect Respiratory Viruses in Persons under Investigation for COVID-19. *J Clin Microbiol*. 2020;59(1). doi:10.1128/JCM.02142-20

10. Barry M, AlMohaya A, AlHijji A, et al. Clinical Characteristics and Outcome of Hospitalized COVID-19 Patients in a MERS-CoV Endemic Area. *J Epidemiol Glob Health*. 2020;10(3):214-221. doi:10.2991/jegh.k.200806.002

11. Blasco ML, Buesa J, Colomina J, et al. Co-detection of respiratory pathogens in patients hospitalized with Coronavirus viral disease-2019 pneumonia. *J Med Virol*. 2020;92(10):1799-1801. doi:10.1002/jmv.25922

12. Brendish NJ, Poole S, Naidu VV, et al. Clinical characteristics, symptoms and outcomes of 1054 adults presenting to hospital with suspected COVID-19: A comparison of patients with and without SARS-CoV-2 infection. *J Infect*. 2020;81(6):937-943. doi:10.1016/j.jinf.2020.09.033

13. Burrel S, Hausfater P, Dres M, et al. Co-infection of SARS-CoV-2 with other respiratory viruses and performance of lower respiratory tract samples for the diagnosis of COVID-19. *Int J Infect Dis*. 2021;102:10-13. doi:10.1016/j.ijid.2020.10.040

14. Calderaro A, Conto F de, Buttrini M, et al. Human respiratory viruses, including SARS-CoV-2, circulating in the winter season 2019-2020 in Parma, Northern Italy. *Int J Infect Dis*. 2021;102:79-84. doi:10.1016/j.ijid.2020.09.1473

15. Castillo EM, Coyne CJ, Brennan JJ, Tomaszewski CA. Rates of coinfection with other respiratory pathogens in patients positive for coronavirus disease 2019 (COVID-19). *J Am Coll Emerg Physicians Open*. 2020. doi:10.1002/emp2.12172

16. Chen S, Zhu Q, Xiao Y, et al. Clinical and etiological analysis of co-infections and secondary infections in COVID-19 patients: An observational study. *Clin Respir J*. 2021;15(7):815-825. doi:10.1111/crj.13369

17. Chen N, Zhou M, Dong X, et al. Epidemiological and clinical characteristics of 99 cases of 2019 novel coronavirus pneumonia in Wuhan, China: a descriptive study. *Lancet*. 2020;395(10223):507-513. doi:10.1016/S0140-6736(20)30211-7

18. Chung H-Y, Jian M-J, Chang C-K, et al. Novel dual multiplex real-time RT-PCR assays for the rapid detection of SARS-CoV-2, influenza A/B, and respiratory syncytial virus using the BD MAX open system. *Emerg Microbes Infect*. 2021;10(1):161-166. doi:10.1080/22221751.2021.1873073

19. Danis K, Epaulard O, Bénet T, et al. Cluster of Coronavirus Disease 2019 (COVID-19) in the French Alps, February 2020. *Clin Infect Dis*. 2020;71(15):825-832. doi:10.1093/cid/ciaa424

20. Clercq J de, Malfait T, Malfait S, et al. Diagnosing COVID-19; towards a feasible COVID-19 rule-out protocol. *Acta Clin Belg*. 2021:1-9. doi:10.1080/17843286.2021.1883362

21. Souza Luna LK de, Perosa AH, Conte DD, et al. Different patterns of Influenza A and B detected during early stages of COVID-19 in a university hospital in São Paulo, Brazil. *J Infect*. 2020;81(2):e104-e105. doi:10.1016/j.jinf.2020.05.036

22. Eisen AKA, Gularte JS, Demoliner M, et al. Low circulation of Influenza A and coinfection with SARS-CoV-2 among other respiratory viruses during the COVID-19 pandemic in a region of southern Brazil. *J Med Virol*. 2021;93(7):4392-4398. doi:10.1002/jmv.26975

23. Elhazmi A, Al-Tawfiq JA, Sallam H, et al. Severe acute respiratory syndrome coronavirus 2 (SARS-CoV-2) and Middle East Respiratory Syndrome Coronavirus (MERS-CoV) coinfection: A unique case series. *Travel Med Infect Dis*. 2021;41:102026. doi:10.1016/j.tmaid.2021.102026

24. Flores-Pérez P, Gerig N, Isabel Cabrera-López M, Unzueta-Roch JL de, Del Rosal T, Calvo C. Acute Bronchiolitis During The Covid-19 Pandemic. *Enferm Infecc Microbiol Clin (Engl Ed)*. 2021. doi:10.1016/j.eimc.2021.06.012

25. Freeman CL, Miller NM, Bastarache L, et al. Co-detection of SARS-CoV-2 with Secondary Respiratory Pathogen Infections. *J Gen Intern Med*. 2021;36(4):1159-1160. doi:10.1007/s11606-020-06471-0

26. Hazra A, Collison M, Pisano J, Kumar M, Oehler C, Ridgway JP. Coinfections with SARS-CoV-2 and other respiratory pathogens. *Infect Control Hosp Epidemiol*. 2020;41(10):1228-1229. doi:10.1017/ice.2020.322

27. Hirotsu Y, Maejima M, Shibusawa M, et al. Analysis of Covid-19 and non-Covid-19 viruses, including influenza viruses, to determine the influence of intensive preventive measures in Japan. *J Clin Virol*. 2020;129:104543. doi:10.1016/j.jcv.2020.104543

28. Hughes S, Troise O, Donaldson H, Mughal N, Moore LSP. Bacterial and fungal coinfection among hospitalized patients with COVID-19: a retrospective cohort study in a UK secondary-care setting. *Clin Microbiol Infect*. 2020;26(10):1395-1399. doi:10.1016/j.cmi.2020.06.025

29. Jongbloed M, Leijte WT, Linssen CFM, van den Hoogen BG, van Gorp ECM, Kruif MD de. Clinical impact of human metapneumovirus infections before and during the COVID-19 pandemic. *Infect Dis (Lond)*. 2021;53(7):488-497. doi:10.1080/23744235.2021.1887510

30. Kim D, Quinn J, Pinsky B, Shah NH, Brown I. Rates of Co-infection Between SARS-CoV-2 and Other Respiratory Pathogens. *JAMA*. 2020;323(20):2085-2086. doi:10.1001/jama.2020.6266

31. Kim KW, Deveson IW, Pang CNI, et al. Respiratory viral co-infections among SARS-CoV-2 cases confirmed by virome capture sequencing. *Sci Rep*. 2021;11(1):3934. doi:10.1038/s41598-021-83642-x

32. Kim Z, Lee JH. Coinfection with severe acute respiratory syndrome coronavirus-2 and other respiratory viruses at a tertiary hospital in Korea. *J Clin Lab Anal*. 2021;35(8):e23868. doi:10.1002/jcla.23868

33. Kıymet E, Böncüoğlu E, Şahinkaya Ş, et al. Distribution of spreading viruses during COVID-19 pandemic: Effect of mitigation strategies. *Am J Infect Control*. 2021;49(9):1142-1145. doi:10.1016/j.ajic.2021.06.002

34. Leuzinger K, Roloff T, Gosert R, et al. Epidemiology of Severe Acute Respiratory Syndrome Coronavirus 2 Emergence Amidst Community-Acquired Respiratory Viruses. *J Infect Dis*. 2020;222(8):1270-1279. doi:10.1093/infdis/jiaa464

35. Li Y, Wang H, Wang F, et al. Co-infections of SARS-CoV-2 with multiple common respiratory pathogens in infected children: A retrospective study. *Medicine (Baltimore)*. 2021;100(11):e24315. doi:10.1097/MD.0000000000024315

36. Lin D, Liu L, Zhang M, et al. Co-infections of SARS-CoV-2 with multiple common respiratory pathogens in infected patients. *Sci China Life Sci*. 2020;63(4):606-609. doi:10.1007/s11427-020-1668-5

37. Ma S, Lai X, Chen Z, Tu S, Qin K. Clinical characteristics of critically ill patients co-infected with SARS-CoV-2 and the influenza virus in Wuhan, China. *Int J Infect Dis*. 2020;96:683-687. doi:10.1016/j.ijid.2020.05.068

38. Marshall NC, Kariyawasam RM, Zelyas N, Kanji JN, Diggle MA. Broad respiratory testing to identify SARS-CoV-2 viral co-circulation and inform diagnostic stewardship in the COVID-19 pandemic. *Virol J*. 2021;18(1):93. doi:10.1186/s12985-021-01545-9

39. Masse S, Bonnet C, Vilcu A-M, et al. Are Posterior Oropharyngeal Saliva Specimens an Acceptable Alternative to Nasopharyngeal Sampling for the Monitoring of SARS-CoV-2 in Primary-Care Settings? *Viruses*. 2021;13(5). doi:10.3390/v13050761

40. Massey BW, Jayathilake K, Meltzer HY. Respiratory Microbial Co-infection With SARS-CoV-2. *Front Microbiol*. 2020;11:2079. doi:10.3389/fmicb.2020.02079

41. Da Matos AR, Motta FC, Caetano BC, et al. Identification of SARS-CoV-2 and additional respiratory pathogens cases under the investigation of COVID-19 initial phase in a Brazilian reference laboratory. *Mem Inst Oswaldo Cruz*. 2020;115:e200232. doi:10.1590/0074-02760200232

42. Mehta P, Sahni S, Siddiqui S, et al. Respiratory Co-Infections: Modulators of SARS-CoV-2 Patients' Clinical Sub-Phenotype. *Front Microbiol*. 2021;12:653399. doi:10.3389/fmicb.2021.653399

43. Nowak MD, Sordillo EM, Gitman MR, Paniz Mondolfi AE. Coinfection in SARS-CoV-2 infected patients: Where are influenza virus and rhinovirus/enterovirus? *J Med Virol*. 2020;92(10):1699-1700. doi:10.1002/jmv.25953

44. Peci A, Tran V, Guthrie JL, et al. Prevalence of Co-Infections with Respiratory Viruses in Individuals Investigated for SARS-CoV-2 in Ontario, Canada. *Viruses*. 2021;13(1). doi:10.3390/v13010130

45. Pigny F, Wagner N, Rohr M, et al. Viral co-infections among SARS-CoV-2-infected children and infected adult household contacts. *Eur J Pediatr*. 2021;180(6):1991-1995. doi:10.1007/s00431-021-03947-x

46. Pongpirul WA, Mott JA, Woodring JV, et al. Clinical Characteristics of Patients Hospitalized with Coronavirus Disease, Thailand. *Emerg Infect Dis*. 2020;26(7):1580-1585. doi:10.3201/eid2607.200598

47. Potdar V, Choudhary ML, Bhardwaj S, et al. Respiratory virus detection among the overseas returnees during the early phase of COVID-19 pandemic in India. *Indian J Med Res*. 2020;151(5):486-489. doi:10.4103/ijmr.IJMR_638_20

48. Richardson S, Hirsch JS, Narasimhan M, et al. Presenting Characteristics, Comorbidities, and Outcomes Among 5700 Patients Hospitalized With COVID-19 in the New York City Area. *JAMA*. 2020;323(20):2052-2059. doi:10.1001/jama.2020.6775

49. Rodriguez C, Prost N de, Fourati S, et al. Viral genomic, metagenomic and human transcriptomic characterization and prediction of the clinical forms of COVID-19. *PLoS Pathog*. 2021;17(3):e1009416. doi:10.1371/journal.ppat.1009416

50. Roh KH, Kim YK, Kim S-W, et al. Coinfections with Respiratory Pathogens among COVID-19 Patients in Korea. *Can J Infect Dis Med Microbiol*. 2021;2021:6651045. doi:10.1155/2021/6651045

51. Schneider JG, Relich RF, Datta D, et al. Identifying Risk Factors That Distinguish Symptomatic Severe Acute Respiratory Syndrome Coronavirus 2 Infection From Common Upper Respiratory Infections in Children. *Cureus*. 2021;13(2):e13266. doi:10.7759/cureus.13266

52. Shah SJ, Barish PN, Prasad PA, et al. Clinical features, diagnostics, and outcomes of patients presenting with acute respiratory illness: A retrospective cohort study of patients with and without COVID-19. *EClinicalMedicine*. 2020;27:100518. doi:10.1016/j.eclinm.2020.100518

53. Si Y, Zhao Z, Chen R, et al. Epidemiological surveillance of common respiratory viruses in patients with suspected COVID-19 in Southwest China. *BMC Infect Dis*. 2020;20(1):688. doi:10.1186/s12879-020-05392-x

54. Singh V, Upadhyay P, Reddy J, Granger J. SARS-CoV-2 respiratory co-infections: Incidence of viral and bacterial co-pathogens. *Int J Infect Dis*. 2021;105:617-620. doi:10.1016/j.ijid.2021.02.087

55. Søgaard KK, Baettig V, Osthoff M, et al. Community-acquired and hospital-acquired respiratory tract infection and bloodstream infection in patients hospitalized with COVID-19 pneumonia. *J Intensive Care*. 2021;9(1):10. doi:10.1186/s40560-021-00526-y

56. Tong X, Xu X, Lv G, et al. Clinical characteristics and outcome of influenza virus infection among adults hospitalized with severe COVID-19: a retrospective cohort study from Wuhan, China. *BMC Infect Dis*. 2021;21(1):341. doi:10.1186/s12879-021-05975-2

57. Wang M, Wu Q, Xu W, et al. *Clinical Diagnosis of 8274 Samples with 2019-Novel Coronavirus in Wuhan*. 2020.

58. Wei L, Gao X, Chen S, et al. Clinical Characteristics and Outcomes of Childbearing-Age Women With COVID-19 in Wuhan: Retrospective, Single-Center Study. *J Med Internet Res*. 2020;22(8):e19642. doi:10.2196/19642

59. Wu C, Chen X, Cai Y, et al. Risk Factors Associated With Acute Respiratory Distress Syndrome and Death in Patients With Coronavirus Disease 2019 Pneumonia in Wuhan, China. *JAMA Intern Med*. 2020;180(7):934-943. doi:10.1001/jamainternmed.2020.0994

60. Xing Q, Li G, Xing Y, et al. *Precautions Are Needed for COVID-19 Patients with Coinfection of Common Respiratory Pathogens*. 2020.

61. Zhu X, Ge Y, Wu T, et al. Co-infection with respiratory pathogens among COVID-2019 cases. *Virus Res*. 2020;285:198005. doi:10.1016/j.virusres.2020.198005

62. Balshem H, Helfand M, Schünemann HJ, et al. GRADE guidelines: 3. Rating the quality of evidence. *J Clin Epidemiol*. 2011;64(4):401-406. doi:10.1016/j.jclinepi.2010.07.015

63. Liberati A, Altman DG, Tetzlaff J, et al. The PRISMA statement for reporting systematic reviews and meta-analyses of studies that evaluate healthcare interventions: explanation and elaboration. *BMJ*. 2009;339:b2700. doi:10.1136/bmj.b2700

64. G. Wells, G. Wells, B. Shea, B. Shea, Dianne O'Connell, J. Peterson, Welch, M. Losos, P. Tugwell, Sb Wells Ga, G. Zello, J. Petersen. The Newcastle-Ottawa Scale (NOS) for assessing the quality of nonrandomised studies in meta-analyses. Accessed March 10, 2022.
